# Foam Cells Control *Mycobacterium tuberculosis* Infection

**DOI:** 10.3389/fmicb.2020.01394

**Published:** 2020-07-09

**Authors:** Pooja Agarwal, Theo W. Combes, Fariba Shojaee-Moradie, Barbara Fielding, Siamon Gordon, Valerie Mizrahi, Fernando O. Martinez

**Affiliations:** ^1^South African Medical Research Council/National Health Laboratory Service/University of Cape Town, Molecular Mycobacteriology Research Unit, Division of Medical Microbiology, Department of Pathology, Department of Science and Innovation/National Research Foundation, Centre of Excellence for Biomedical TB Research and Wellcome Centre for Infectious Diseases Research in Africa, Institute of Infectious Disease and Molecular Medicine, University of Cape Town, Cape Town, South Africa; ^2^Faculty of Health and Medical Sciences, University of Surrey, Guildford, United Kingdom; ^3^Graduate Institute of Biomedical Sciences, College of Medicine, Chang Gung University, Taoyuan City, Taiwan; ^4^Sir William Dunn School of Pathology, University of Oxford, Oxford, United Kingdom

**Keywords:** tuberculosis, foam cells, macrophage, lipid droplets, cytokines, inflammation

## Abstract

*Mycobacterium tuberculosis* (*Mtb*) infects macrophages and macrophage-derived foam cells, a hallmark of granulomata in tuberculous lesions. We analyzed the effects of lipid accumulation in human primary macrophages and quantified strong triglyceride and phospholipid remodeling which depended on the dietary fatty acid used for the assay. The enrichment of >70% in triglyceride and phospholipids can alter cell membrane properties, signaling and phagocytosis in macrophages. In conventional macrophage cultures, cells are heterogeneous, small or large macrophages. In foam cells, a third population of 30% of cells with increased granularity can be detected. We found that foam cell formation is heterogenous and that lipid accumulation and foam cell formation reduces the phagocytosis of *Mtb*. Under the conditions tested, cell death was highly prevalent in macrophages, whereas foam cells were largely protected from this effect. Foam cells also supported slower *Mtb* replication, yet this had no discernible impact on the intracellular efficacy of four different antitubercular drugs. Foam cell formation had a significant impact in the inflammatory potential of the cells. TNF-α, IL-1β, and prototypical chemokines were increased. The ratio of inflammatory IL-1β, TNF-α, and IL-6 vs. anti-inflammatory IL-10 was significantly higher in response to *Mtb* vs. LPS, and was increased in foam cells compared to macrophages, suggestive of increased pro-inflammatory properties. Cytokine production correlated with NF-κB activation in our models. We conclude that foam cell formation reduces the host cell avidity for, and phagocytosis of, *Mtb* while protecting the cells from death. This protective effect is associated with enhanced inflammatory potential of foam cells and restricted intracellular growth of *Mtb*.

## Introduction

Foam cell macrophages have been described in varied human pathologies from the early twentieth-century. The name was attributed to the foamy appearance of cells, with cytoplasm filled with different size lipid droplets and vesicles. Anitschkow, a Russian pathologist (1885–1964), discovered the accumulation of cholesterol esters in myocardial macrophages and smooth muscle myocytes in atherosclerosis and developed a widely used rabbit experimental model to study its pathogenesis, as reviewed by Steinberg (2013). Our knowledge about the composition and origin of lipids that accumulate in foam cell macrophages and their effects in immunity remain poorly defined.

Foam cells appear in a variety of conditions where macrophages need to dispose of excessive lipids, and these can be of different nature (Chistiakov et al., 2017; Guerrini and Gennaro, 2019). Atherosclerosis is not their only arena and foam cells are a particular feature of tuberculous infection and caseation, a hallmark of persistent mycobacterial immune reactions (Peyron et al., 2008; Jaisinghani et al., 2018). Foam cells are abundant in mycobacterial granulomata, and the general assumption is that these cells accumulate lipids and die, leading to caseation which contributes to chronic inflammation, tissue damage and dissemination of the pathogen (Hunter et al., 2007; Kim et al., 2010; Guerrini and Gennaro, 2019). A role for IFN-γ in this process has been shown, as well as for lipids derived from the host and the bacterium (Knight et al., 2018).

Fatty acids are abundant in serum and tissues, in esterified form in lipid classes such as phospholipids and triglyceride (TG), or in non-esterified form in serum, interstitial fluid, or in cells. Their composition can be modulated by diet (Laganiere and Byung, 1993; Hodson et al., 2008; Khadge et al., 2018) or endogenous synthesis. Phospholipids are typically found in membranes, or surrounding lipoproteins, whereas TGs are found in lipid droplets, or transported in the blood in the core of lipoproteins. Fatty acyl-containing lipid species in the milieu lead to their accumulation in lipid droplets in macrophages as recently reviewed (Remmerie and Scott) (Remmerie and Scott, 2018). This is relevant in *Mycobacterium tuberculosis* (*Mtb*) where biochemical analyses of lipids within the caseum identified TGs, cholesterol, cholesteryl esters, lactosylceramide, implicating lipids derived from low density lipoprotein as a likely source (Kim et al., 2010). More recently, the accumulation of TGs in tuberculous lung lesions mediated by a TNF-α receptor-mediated signaling mechanism, which is distinct from that leading to intracellular cholesteryl ester accumulation in atherosclerosis, highlights the disease specificity of foam cell biogenesis (Guerrini et al., 2018; Guerrini and Gennaro, 2019).

Characterizing the role of fatty acid supplementation in the immune response to *Mtb* opens an important opportunity for nutritional intervention. In this paper, we investigate the effect of exogenous fatty acid accumulation in human macrophages and THP-1-derived macrophages, on the outcome of *Mtb* infection with a focus on the phagocytic reaction, and the host response elicited by the bacterium.

## Materials and Methods

### Bacterial Strains and Growth Conditions

Wild type *Mtb*-H37Rv (H37RvMA; Ioerger et al., 2010) or *Mtb*-H37Rv:mCherry (*Mtb-*H37Rv carrying a vector expressing mCherry; Carroll et al., 2010) were grown at 37°C in Middlebrook 7H9 medium (Difco^TM^), supplemented with 10% v/v oleic acid albumin dextrose-catalase (OADC) (Difco^TM^), 0.5% tween 80 (Sigma), and 0.02% v/v glycerol (Sigma) with or without 50 μg/ml hygromycin B (Sigma) as indicated in the text. Bacteria were grown to mid-exponential phase (OD_600_ ∼ 0.5).

### Isolation and Culture of Primary Human Monocytes and Macrophages

Human monocytes were isolated from healthy blood donors. Small fresh blood donations of 50 ml or less were acquired in the laboratory under ethics approval (UEC/2017/052/FHMS), and leukocyte cones were acquired from NHSBT blood services under ethics permit REC (17/LO/1877). Where indicated, monocytes were isolated with Ficoll and Percoll gradients as previously described (Martinez, 2012) (GE Healthcare), or Ficoll and CD14 positive isolation according to manufacturer’s instructions (Miltenyi). To generate loosely adherent macrophages, monocytes were cultured in hydrophobic 35 mm lumox culture dishes (Sarstedt) at a density of 3 × 10^5^ cells/cm^2^ and cell/volume ratio of 1 × 10^6^ cells/ml. To generate firmly adherent macrophages, monocytes were seeded at a density of 3 × 10^5^ cells/cm^2^ and cell/volume ratio of 1 × 10^5^ cells/ml in tissue culture plastic (Nunc^TM^). For maturation, we used Opti-MEM media (Life Technologies) supplemented with 1% heat-inactivated pooled (non-autologous) human serum (Sigma); cells were cultured at 37°C in a humidified 5% CO_2_ incubator for 7 days unless specified.

### Culture of THP1-Derived Macrophages

Human promonocytic THP-1 cells (ATCC) were maintained in RPMI-1640 medium (Sigma) with 2 mM L-glutamine supplemented with 10% fetal bovine serum (Gibco) at 37°C in a humidified, 5% CO_2_ atmosphere. Cell line maintenance was performed according to supplier instructions. Maturation of THP-1 into macrophages was induced by incubation with 200 nM of phorbol 12-myristate 13-acetate (PMA; Sigma) for 24 h.

### Foam Cell Formation

For foam cell induction, primary 1% human-serum-derived monocyte-derived macrophages were exposed after the third day of maturation to varying concentrations of the following fatty acids (Sigma): α-linoleic acid-oleic acid-albumin, α-linoleic acid-albumin or oleic acid-albumin. Fatty acid incubation was performed for 96 h unless indicated otherwise. For foam cell induction in THP-1 cells, 24 h, PMA-treated THP-1 macrophages were exposed to the fatty acids for 72 h unless indicated otherwise. The accumulation of lipid droplets was confirmed by staining neutral lipids with Nile Red (Sigma) or LipidTOX (Thermo Fisher).

### Triglyceride and Phospholipid Analysis in Whole Cells

For lipid analysis, 2 × 10^6^ macrophages or foam cells were harvested in 500μl calcium and magnesium-free PBS in glass tubes. Total lipids were extracted using 2:1 (v:v) chloroform:methanol containing 0.01% (% w/v) butylated hydroxytoluene as preservative (Folch et al., 1957). Lipid classes were separated by thin layer chromatography using 20 × 20 cm aluminum sheets coated with Silica Gel 60 (VWR International Ltd.) (Fielding et al., 1996). Lipid spots were visualized with 0.1% (w/v) solution of 8-anilino-1-naphthalenesulfonic acid in water and identified using TLC standards (Sigma). Each sample spot was scraped into a glass tube and fatty acid methyl esters prepared by adding 400 μl toluene and 800 μl of 6% H_2_SO_4_ in methanol in a heating block at 80°C for 2 h. The fatty acid methyl esters were extracted into hexane and evaporated to dryness before reconstituting into 200 μl hexane (Burdge et al., 2000). Individual fatty acids were quantified by gas chromatography (GC)-mass spectrometry (MS) using an Agilent system comprising a 7683 GC equipped with a 30 m, 250 μm internal diameter, 0.25 μm film thickness FAMEWAX capillary column –Crossbond^TM^ polyethylene glycol (Thames Restek) coupled to a 5973-network mass selective detector (Agilent Technologies). The injector temperature was 250°C. Helium was used as the carrier gas at a flow rate of 1.3 mL/min. The initial GC oven temperature was 100°C, which was held for 3 min, then increased to 200°C at a rate of 25°C/min and held for 6 min. This was then increased to a final temperature of 240°C and held for 5 min. Individual FAME peaks were identified based on their retention time relative to the analytical standard RM6 (FAMEs mixture; Supelco), as well as by comparing their mass spectra to an online standard reference database (National Institute of Standards and Technology; NIST).

### Phagocytosis of *Mtb*-H37Rv

The phagocytic reaction toward *Mtb* was studied using *Mtb*-H37Rv grown to logarithmic phase. The bacteria were killed by fixation with 4% paraformaldehyde and labeled with fluorescein isothiocyanate (FITC; Sigma) according to the manufacturer’s instructions. Macrophages or foam cells were exposed to fixed, FITC-stained bacteria at multiplicity of infection (MOI) of 10:1 (bacilli per macrophage), for indicated time points. Macrophages were subsequently stained with Zombie-NIR fixable viability dye (Biolegend) and cells were acquired on a BD Celesta flow cytometer. The analysis flowchart included single cell and live cell selection as indicated in the text. Compensation was carried out using single stained cells. Quantification of events was done with Precision Count Beads^TM^ (Biolegend) using the formula:

(1) Absolute Cell Count (cells/µl)=Cell Count ×Precision Count BeadsTM VolumePrecision Count BeadsTM Count×Cell Volume× Precision Count BeadsTM Concentration

### Infection of Cells With Live *Mtb*-H37Rv

Human foam cells or macrophages were infected with live, logarithmic-phase *Mtb*-H37Rv at a MOI of 5:1 (bacilli per macrophage) for 4 h. Extracellular bacteria were removed with PBS washes. The inoculum dosage was confirmed by plating on Middlebrook 7H10 agar (Difco) for colony-forming unit (CFU) enumeration. THP-1 and THP1-Lucia^TM^ foam cells or resting cells were infected with either wild type *Mtb*-H37Rv or *Mtb*-H37Rv:mCherry at a MOI of 5:1 for 4 h.

### Intracellular Growth and Drug Susceptibility Testing of *Mtb*-H37Rv

To compare the growth kinetics of *Mtb*-H37Rv in foam cells vs. macrophages, *Mtb*-infected cells sampled at selected time points over a 7-day time course were lysed with 0.05% Triton X-100, and dilutions of the lysates plated onto Middlebrook 7H10 agar supplemented with 5% glycerol and 10% OADC for CFU enumeration. Bacterial colonies were counted 3–5 weeks after plating. Intracellular growth of *Mtb*-H37Rv:mCherry in oleic-acid-induced THP-1 foam cells was also monitored by measuring fluorescence of the mCherry reporter on days 1, 3, 5, and 7 using a Fluostar plate reader (BMG Biotech) at excitation and emission wavelengths of 587 and 601 nm, respectively. On selected days, images of the infected cells were acquired with ZOE^TM^ Fluorescent Cell Imager (Bio-Rad) and compared with CFU enumeration. *Mtb*-infected THP-1 foam cells or resting macrophages were exposed to 0.05 μg/ml rifampicin, 0.5 μg/ml isoniazid, 0.8 μg/ml moxifloxacin or 3.1 μg/ml bedaquiline (Sigma). For CFU enumeration, cells were lysed on days 0, 2, 4 and 6, serial dilutions of lysates were plated on 7H10 agar, and colonies counted after 3–5 weeks.

To assess the effect of anti-TB drug treatment on host cell viability, uninfected foam cells and resting cells were treated with each drug at the same concentration as used in the intracellular drug susceptibility assays described above. At days 1 (i.e., 24 h post-treatment), 2, 4, and 6, cells were washed three times and viability assessed by adding 10% (v/v) of the vital stain, Alamar Blue, which incorporates a redox indicator that fluoresces and changes color through chemical reduction as a consequence of cell growth. Fluorescence was measured after 5 h using a Fluostar plate reader (BMG Biotech) at an excitation wavelength of 560 nm and emission wavelength of 590 nm.

### THP1-Lucia^TM^ NF-κB Luciferase Reporter Assay

THP1-Lucia^TM^ NF-κB cells (Invivogen) were cultured in a comparable manner to wild type THP-1 cells to generate macrophages and foam cells. THP1-Lucia^TM^-derived foam cells were infected with *Mtb*-H37Rv for 4 h, then washed with PBS to remove extracellular bacteria. Cell culture supernatants were collected at 24 h post-infection and filtered twice through 0.22 μm centrifuge tube filters (Sigma). To measure luciferase activity, 10 μL of the supernatants were transferred to white-bottom 96-well plates and monitored for activity in the presence of 2 μg/mL of coelenterazine substrate (NanoLight Technology) with a Clariostar plate reader (BMG Biotech).

### Cytokine and Chemokine Quantification Assay

To quantify cytokine and chemokine secretion, supernatants were collected and sterilized using 0.22 μm filters and stored at -80°C until analyzed. The production of the chemokines CCL2, CCL5, CXCL8, CXCL9, CXCL10, and the cytokines IL-1β, TNF-α, IL-6, IL-10, IL-12 p70, by THP-1 derived macrophages and foam cells, was quantified using a Cytometric Bead Array kit (BD Biosciences) according to the manufacturer’s instructions.

TNF-α production in culture supernatants from THP1-Lucia^TM^ NF-κB cells derived macrophages and foam cells was quantified using the Human TNF-α ELISA (eBioscience or Invitrogen) according to the manufacturer’s instructions.

### Confocal Microscopy Acquisition and Analysis

The presence of lipid droplets in human macrophages and foam cells was confirmed microscopically using the EVOS FL Cell Imaging System. Cells were washed with PBS and stained with green LipidTOX (Invitrogen) and Hoechst (Invitrogen) as per the manufacturer’s instructions. THP-1 cells were washed with PBS and stained with LipidTOX and DAPI (Sigma) as per the manufacturers’ instructions and processed for confocal microscopy in a Zeiss LSM 880 with Fast Airyscan module confocal microscope (Carl Zeiss, Jena, Germany). LipidTOX (green) was detected with the Argon 488 nm laser, mCherry (red) with the 561 nm laser, and DAPI (blue) with the 405 nm laser using a Plan Apochromat 63X/1.4 NA Oil DIC objective. Images were processed using ZEN software.

## Results

### Foam Cells Can Be Tailored by Fatty Acid Supplementation

To investigate the contribution of specific fatty acid supplementation to the triglyceride and phospholipid pool in foam cells we exposed 1% human serum macrophages on the third day of culture, to 100, 200, or 400 μM of oleic acid (18:1 n-9) or linoleic acid (18:2 n-6), or a mixture of both (each at a concentration of 400 μM). After 4 days cells were stained with Nile Red for neutral lipid detection and DAPI for nuclei and inspected in the microscope.

**FIGURE 1:**
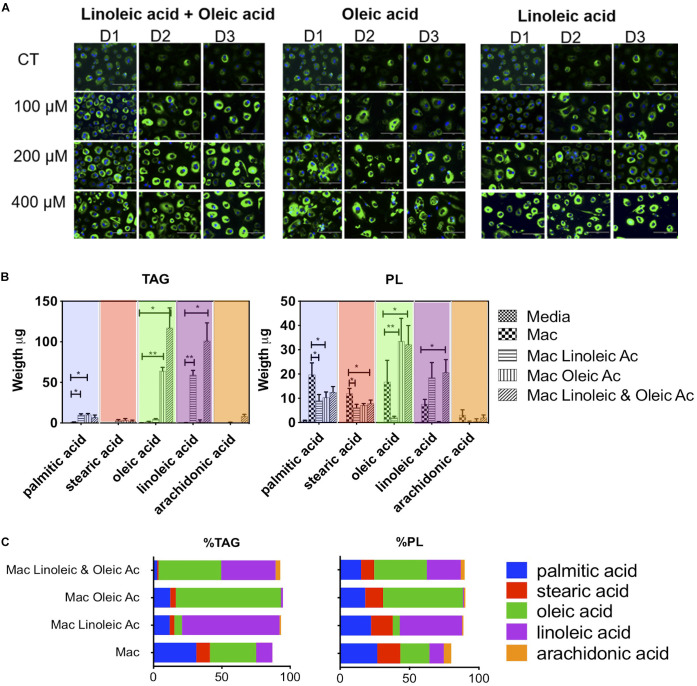
Lipid composition of human macrophage can be tailored by exposing them to specific fatty acids. **(A)** Human monocytes from 3 donors (D1, D2, and D3) were matured into macrophages in the presence of 1% human serum (*n* = 3). At day 3, macrophages were exposed to increasing concentrations of oleic acid, linoleic acid, a combination of both or left untreated (CT). The treatment was maintained for 4 days. At day 7, cells were stained with Nile Red to assess the formation of lipid droplets. **(B)** Quantification and proportion of fatty acids detected in TG and PL of macrophages exposed to fatty acid (400 μM of each). The figure shows the weight of fatty acids in triglycerides and phospholipids detected after 4 days of lipid exposure, *n* = 3. We performed a two-way ANOVA and multiple comparisons were made using the macrophage model as control. The two-way ANOVA yielded a *p* < 0.0001. Inter treatment differences with *p* < 0.005 are indicated in the graph with an asterisk **(C)** Proportion of fatty acids in each cell isolate. The percentage was calculated in each donor considering the total TAG, PL and individual fatty acid abundance. The average of *n* = 3 donors is plotted. *p* values, 0.0332(*), 0.0021 (**), 0.00002 (***), 0.0001 (****).

Microscopically we found neutral lipid droplets accumulation in human-serum-derived macrophages, which was exacerbated when cells were cultured in the presence of oleic acid (18:1 n-9), the essential fatty acid linoleic acid (18:2 n-6) or a mixture of both fatty acids (Figure 1A). A similar lipid droplet accumulation upon exposure to fatty acids was observed in macrophages derived from the monocytic cell line THP-1 (Supplementary Figure S2). In these models, foam cells retained lipid droplets for up to 6 days (Supplementary Figure S3). Macrophages showed no signs of toxicity upon exposure to oleic acid at concentrations between 100μM and 400μM as assessed by fluorescent cell imaging with Calcein-AM and EthD1 (Supplementary Figure S1).

We quantified the weight in μg of each fatty acid in triglycerides and phospholipids in a total of 2 × 10^6^ cells (Figure 1B). Interestingly, whilst lipid droplets were detected microscopically in serum macrophages, these cells do not show a substantial accumulation of triglycerides; we detected an average of 3.7 μg of triglycerides per cell isolate. Triglyceride synthesis and accumulation was only exacerbated by fatty acid treatment. Exposure to 400 μM linoleic acid or oleic acid induced an accumulation of 77 or 79 μg of triglycerides per cell isolate, respectively. The combination of linoleic and oleic acids, each at 400 μM, drove accumulation of an average of 236 μg of triglycerides per isolate. Therefore, while oleic acid or linoleic acid alone at a concentration of 400 μM drove a ∼20-fold increase in triglyceride content, a synergistic increase in triglyceride content (64-fold) was induced by supplementation with a combination of the two fatty acids, each at 400 μM.

In the triglyceride pool of serum matured macrophages (total 3.7 μg), we found the following fatty acid distribution: 34% oleic acid, 31% palmitic acid, 12% linoleic acid, 10% stearic acid, and no arachidonic acid (18:4 n-6) (Figure 1C). Linoleic acid treatment increased linoleic acid representation from 12 to 70% of total triglycerides (linoleic acid, 59 μg out of 77 μg). Oleic acid treatment increased oleic acid in the cells from 34 to 77% of total triglycerides (oleic acid, 64 μg out of 79 μg). The combination of both fatty acids led to accumulation of 40 and 46% of linoleic acid or oleic acid, respectively, in triglycerides (oleic acid, 117 μg, and linoleic acid, 102 μg out of a total of 236 μg).

Fatty acid exposure had milder and different effects on the total level of phospholipids detected, with a decrease from 59 μg per isolate in human serum macrophages to 36 and 51 μg for linoleic acid and oleic acid, respectively. The combination of fatty acids with twice the molarity induced an increase in phospholipids from 59 to 75 μg. In the phospholipid fraction of serum macrophages, we found an average 27% of palmitic acid, 21% oleic acid, 17% stearic acid, 10% of linoleic acid and 5% of arachidonic acid (Figure 1C). In phospholipids, linoleic acid exposure led to an increase from 10 to 45% and oleic acid exposure from 21 to 58%. In the combination of linoleic acid and oleic acid, each fatty acid increased to 24 and 38% respectively. Arachidonic acid, which is biologically significant was minimally changed or detected in this setting.

The changes observed in fatty acid composition have the potential to alter cell membrane properties, signaling and phagocytosis in macrophages. We therefore investigated whether fatty acid supplementation alters the innate immune reaction between macrophages, foam cells and *Mtb*.

### Oleic Acid-Induced Human Macrophage Foam Cells Have Impaired *Mtb* Phagocytosis

We first compared the ability of human monocyte derived macrophages and oleic-acid-induced foam cells to phagocytose *Mtb*-H37Rv. In this assay, macrophages were cultured from blood monocytes for 7 days. Half of the cells were stimulated at day 3 with 400 μM oleic acid to promote foam cell formation.

**FIGURE 2:**
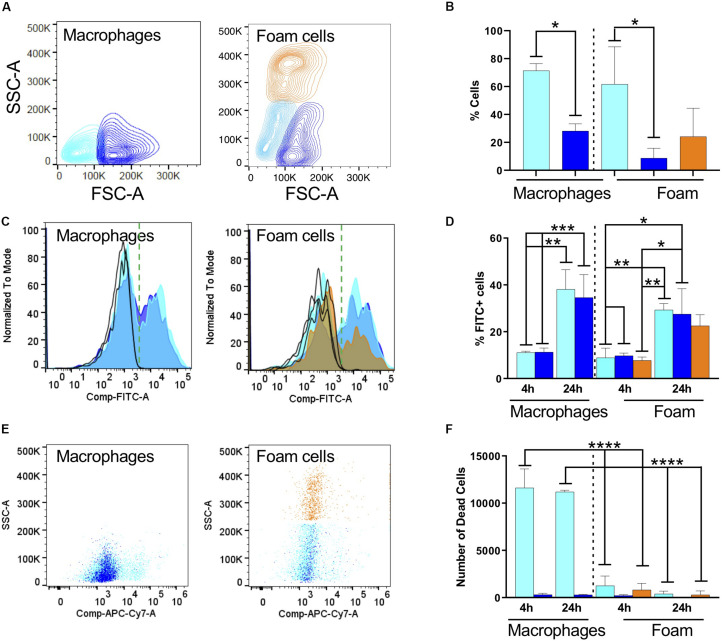
Fatty acid exposure alters the host cell response to infection with fixed, FITC-stained *Mtb*. **(A)** Representative contour plots of side scatter area (SSC-A) vs. forward scatter area (FSC-A) for macrophages and **(B)** oleic-acid-derived foam cells. The cell populations were subdivided according to size and granularity: cyan, small macrophage; blue, large macrophages; and orange, granular foam cells. The same color code is used in the rest of the figure. **(C)** Representative histograms for phagocytosis of *Mtb*-H37Rv after 24 h indicated as FITC positivity for macrophages and foam cells. We recovered different numbers of cells from macrophages or foam cells. To focus on FITC positivity, samples were made comparable by normalizing to the mode of the number of cells acquired and representing the % as is customary in FACS analysis. Due to using live/dead staining for macrophage markers (CD64 and CD40, not shown), the FITC channel was compensated and this is indicated in the axis as Comp-FITC. **(D)** Quantification of *Mtb* (FITC) phagocytosis expressed as a percentage of FITC-positive cells. **(E)** Representative scatter plots of cell viability, 24 h post-phagocytosis, measured as incorporation of Zombie dye. **(F)** Quantification of the number of dead cells using Biolegend Precision Count Beads after 4 h and 24 h. Statistical analysis for all panels was performed with one or two-way ANOVA followed by Tukey’s multiple comparison *t*-test; *p < 0.05, **p = 0.0021, ***p = 0.0002, and ****p = < 0.0001. All experiments were performed in 3 individual donors and mean with SD was plotted for each measurement.

With cytometry analysis we found that macrophages and foam cells have strikingly different size and granularity profiles (Figures 2A,B). Briefly, we separated cells according to their forward scatter indicative of size and the side scatter indicative of granularity and cytoplasm complexity. In human serum macrophages we found that *ca*. 70% of macrophages are small (color-coded cyan), whereas 30% of the cells acquire a large macrophage phenotype (color-coded blue). The ratio between these phenotypes was not altered by exposure to *Mtb*. The size and granularity distribution changes dramatically upon fatty acid exposure and foam cell formation: 30% of the cells became highly granular (color-coded orange), which we associate with maximal lipid accumulation and droplet formation (Figures 2A,B). The remaining cells remain small or large, approximately 50% and 20% respectively. Both small and large cells showed a moderate increase in granularity, as seen in the upward shift in the side scatter, but not comparable to the orange population.

We exposed macrophages and foam cells to formaldehyde-fixed FITC-stained *Mtb*-H37Rv for 4 or 24 h (Figures 2C,D). FACS analysis of cells positive for *Mtb*, represented in Figure 2 as a histogram of the FITC channel, showed a bimodal distribution, with cells negative for FITC which did not phagocytose the bacterium, and cells positive for FITC which did phagocytose the bacterium. In serum-derived cells, 10% of the small and large macrophages became positive for *Mtb* after 4 h, and this proportion increased to 40% after 24 h exposure (Figure 2D and Supplementary Figure S5). The median fluorescence intensity (MFI) did not change which indicates that the cells phagocytosed similar numbers of bacteria. The normalized counts of cells positive for the bacterium are comparable irrespective of cell size indicating that similar numbers of macrophages phagocytosed *Mtb*.

In foam cells, we found a similar bimodal distribution, and again comparable MFIs, suggestive of the fact that all cells phagocytosed the same number of bacteria. However, we could observe differences in the number of cells which phagocytosed. The histogram in orange, representative of the most granular foam cells, shows a significant decrease in the number of cells positive for the bacterium, corresponding to a *ca*. 20% drop compared to the serum derived macrophage phagocytic levels. Although difficult to compare directly across models due to size variations, these results suggest a consistently lower phagocytosis rate of *Mtb* by foam cells. This can be seen by a decrease in the orange bar, in both the inter- and intra- comparisons. These measurements were corroborated in foam cells induced with a lower dose of oleic acid (*n* = 3, 40 μM; data not shown).

The FACS analysis was also used to measure cell death, a prototypic hallmark of the reaction of macrophages to *Mtb* and the main suspect behind caseation which leads to dissemination of infection. We introduced Precision Count Beads to quantify the output of dead cells vs. the input. *Mtb* fixation did not inhibit cell death in our model. Under the conditions tested, cell death was highly prevalent in macrophages, whereas foam cells were largely protected from this effect (Figures 2E,F).

Together, these results suggested that oleic-acid-induced foam cells phagocytose fewer bacteria and exhibit less cell death compared to control macrophages in response to infection with dead *Mtb*-H37Rv.

### Foam Cells Are Restrictive for *Mtb* Growth

**FIGURE 3:**
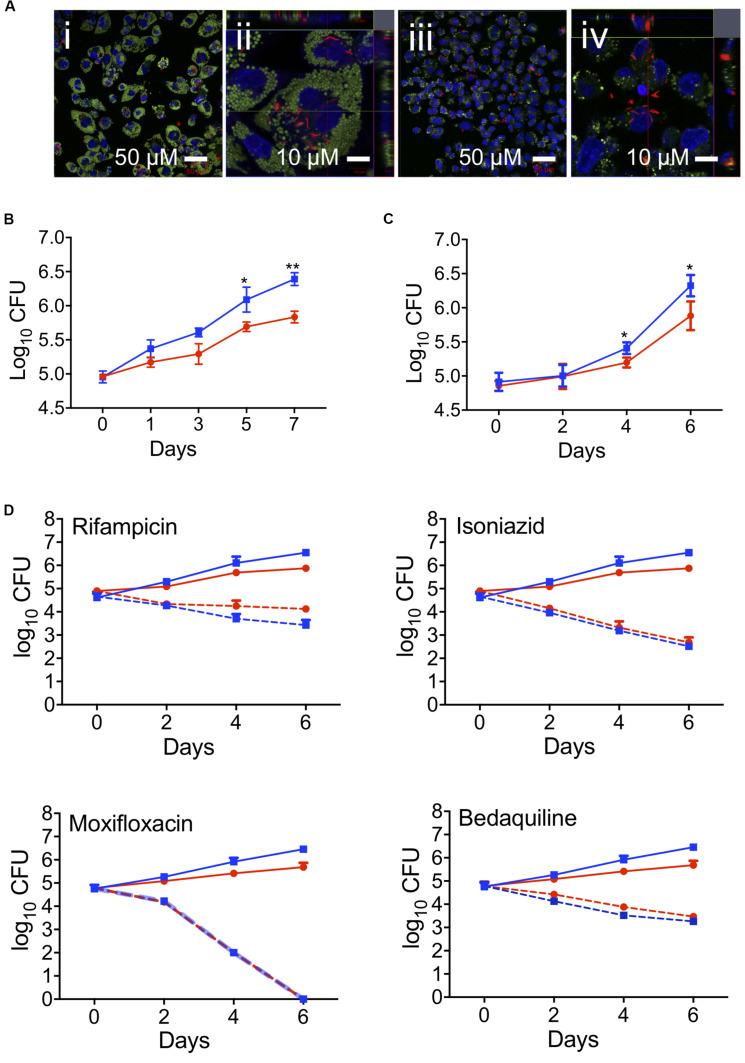
Effect of foam cell formation on intracellular growth of *Mtb* and the time-kill kinetics of anti-TB drugs. **(A)** Oleic-acid-induced THP-1-derived foam cells or resting macrophages were infected with *Mtb*-H37Rv:mCherry and processed for confocal microscopy as described in section “Materials and Methods.” Merged confocal images show the infection of foam cells (i and ii) or resting macrophages (iii and iv) by *Mtb*-H37Rv:mCherry. Lipid droplets were stained green with LipidTOX, nucleic stained blue with DAPI, and bacilli are shown in red. Scale bar is 50 μm (i and iii) or 10 μm (ii and iv). **(B)** Growth of *Mtb* in oleic-acid-induced THP-1-derived foam cells vs. resting macrophages, as determined by CFU enumeration. The data shown are the average ± SD from a representative experiment (*n* = 3); *p*-values were calculated by unpaired *t*-test, *p = 0.0245, **p = 0.0017. **(C)** Growth of *Mtb*-H37Rv in human macrophages exposed to a mixture of linoleic and oleic acid vs. resting macrophages. The data are representative of experiments performed on the samples from three donors; *p = 0.0216. **(D)** Oleic-acid-induced THP-1-derived foam cells and resting cells infected with *Mtb*-H37Rv were exposed to drug at a concentration 10 × above the *in vitro* MIC_90_. The data show the average ± SD from a representative experiment (*n* = 3). Blue squares, untreated resting macrophages; red circles, untreated foam cells; blue dotted squares, drug-treated resting macrophages; red dotted circles, drug-treated foam cells.

The phagocytic capacity of foam cells for live *Mtb* was then assessed by confocal microscopy using THP-1-derived macrophages and oleic acid (400 μM)-induced THP-1 foam cells infected with *Mtb*-H37Rv:mCherry for 4 h at a MOI of 5 (bacteria per cell) (Figure 3A). Most of the mCherry-expressing bacteria were found to be associated with the foam cells (Figure 3Ai). Z-stack imaging of the infected foam cells showed that the bacteria were located within the foam cells rather than adhering to the surface (Figure 3Aii), thus confirming that foam cells can indeed phagocytose live *Mtb*, as observed for the resting macrophage control (Figures 3Aiii,iv).

To determine the effect of foam cell formation on the intracellular growth of *Mtb*, THP-1-derived macrophages and foam cells were infected with *Mtb* as described above and monitored for bacillary growth over 7 days by CFU enumeration (Figure 3B) and fluorescence-based readout (Supplementary Figure S4B). In both cell types, the bacterial load increased progressively over 7 days, however, *Mtb* growth was delayed in foam cells which also acquired a significantly lower bacterial burden at day 7 (0.5log_10_) than resting macrophages (Figure 3B). The impaired growth of *Mtb* in foam cells, as determined by CFU enumeration, was corroborated by fluorescence readout (Supplementary Figure S4A). Representative images of the same cell population used to monitor the fluorescence readout showed a progressive increase in fluorescence intensity in foam cells and resting cells, confirming replication of *Mtb* in both cell-types albeit more slowly in foam cells.

*Mtb* growth was likewise restricted in foam cells produced by exposure of macrophages cultured from fresh monocytes to a mixture of linoleic acid and oleic acid (400 μM each) prior to infection with *Mtb*-H37Rv for 4 h at a MOI of 5 (bacteria per cell). Here, cells were isolated with Ficoll and Percoll gradient. In this model, *Mtb* growth was negligible until day 2; thereafter, bacillary growth was observed in both cell types, albeit more slowly in foam cells than resting macrophages (Figure 3C). At day 6, the bacillary load in foam cells was 0.6log_10_ lower than in macrophages (5.7log_10_ vs. 6.3log_10_).

These results confirmed triglyceride-enriched foam cells produced from human monocyte derived macrophages by supplementation with a mixture of linoleic acid and oleic acid, or THP-1-derived macrophages produced by supplementation with oleic acid alone, both supported slow *Mtb* replication.

### Foam Cell Formation Does Not Affect the Efficacy of Anti-TB Drugs

First- and second-line anti-TB drugs can accumulate in macrophages and may be affected by lipids (Sarathy et al., 2017, 2018; Schump et al., 2017; Blanc et al., 2018; Greenwood et al., 2019). To evaluate the effect of foam cell formation on antitubercular drug efficacy, we exposed oleic-acid-induced THP-1 foam cells and resting macrophage controls, infected with *Mtb*-H37Rv, to drug at a concentration 10-fold higher than that required to inhibit the growth of 90% of the organisms in standard *in vitro* culture (MIC_90_) and monitored the effect on bacillary load by plating cell lysates for CFU enumeration after 0, 2, 4, and 6 days of drug treatment.

At the concentrations tested, none of the drugs was toxic to macrophages, as determined using an Alamar Blue assay (Estrella et al., 2011; Supplementary Figure S6). However, all four drugs showed significant bactericidal activity against *Mtb* over the 6-day time course in both resting and foam cell macrophages with isoniazid, moxifloxacin and bedaquiline demonstrating indistinguishable efficacy in both cell types. Rifampicin appeared to be moderately less efficacious in foam cells, however, the difference was not statistically significant (Figure 3D). Therefore, despite the slower rate of replication of *Mtb* observed in the lipid droplet-laden foam cells, this had no discernible impact on the intracellular efficacy of the four anti-TB drugs under the conditions tested.

### The Balance of NF-κB Innate Inflammatory Responses Is Altered in Foam Cells

**FIGURE 4:**
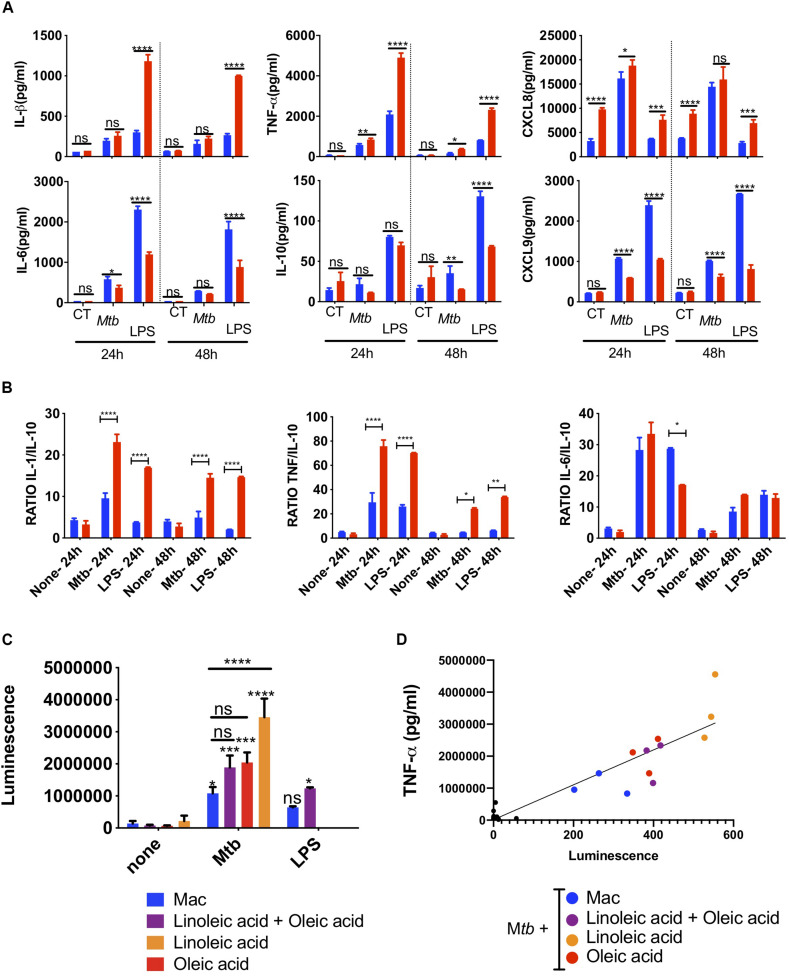
Innate super-activation in lipid laden cells. Comparative cytokine and chemokine secretion by oleic-acid-induced THP-1 foam cells vs. resting macrophages infected with *Mtb*-H37Rv. LPS (100 ng/ml) was used as a positive control. Culture supernatants from *Mtb*-infected, LPS-exposed or uninfected and unexposed control cells (CT) were collected 24 h and 48 h after *Mtb* infection or LPS exposure and analyzed by flow cytometry using a cytometric bead array kit. **(A)** The top row includes examples of cytokines and chemokines secreted at significantly higher levels by foam cells (red bars) compared to resting macrophages (blue bars), whereas the bottom row shows the converse. **(B)** The ratio between inflammatory IL-1β, TNF-α, and IL-6 and anti-inflammatory IL-10 is shown in the graph. Statistical differences were assessed with two-way ANOVA, and Sidak’s was used as multiple comparisons test. The ANOVA analysis showed *p*-values for both treatment and dose < 0.0001. Statistically significant differences between foam cells (red bars) and macrophages (blue bars) for each stimulus are indicated with an asterisk: *p < 0.05, **p < 0.01, ***p < 0.001, ****p < 0.0001. **(C)** *Mtb* induces NF-κB-activation in foam cells. THP-1-Lucia^TM^ foam cells were induced with 400 μM of lipid [oleic acid or linoleic acid alone, or an equimolar mixture of both (400 μM each)], as indicated. LPS (50 ng/ml) was used as positive control for NF-κB activation. Culture supernatants were collected and used to monitor NF-κB activation by measuring luciferase activity, as described in section “Materials and Methods.” **(D)** TNF-α secretion in culture supernatant was measured by ELISA and the data were correlated to reporter luminescence. Black dots close to the intersection between the *x* and *y*-axis points correspond to lipid-only treated cells not infected with *Mtb*. Data are represented as average ± SD from a representative experiment (*n* = 6), *p < 0.05, **p < 0.01, ***p < 0.001, ****p < 0.0001 by one-way ANOVA with Bonferroni post-tests.

Focusing on the oleic-acid-induced THP-1 foam cell model, we analyzed the secretion of inflammatory cytokines in response to *Mtb* infection. THP1 macrophages produced high levels of TNF-α and IL-1β upon *Mtb* infection (Figure 4A). TNF-α secretion was exacerbated in a statistically significant manner in oleic acid derived foam cells at 24 and 48 h post-infection. IL-1β was also increased albeit without statistical significance.

Chemokines amplify the local immune response. In *Mtb*-infected foam cells, we also found increased secretion of the neutrophil chemoattractant, CXCL8 (Figure 4A), CCL2, CCL5, and CXCL10 (Supplementary Figure S7). Conversely, IL-6 and IL-10 were higher in macrophages than foam cells; both can act as anti- and pro-inflammatory cytokines. The T-cell chemoattractant CXCL9 was also secreted at a lower level in foam cells at 24 and 48 h.

We then compared the effects of *Mtb* with those induced by the TLR4 ligand, lipopolysaccharide (LPS, O111:B4) derived from *E coli*. With the exception of CXCL8, LPS used at 100 ng/ml, induced much higher levels of cytokines than whole *Mtb*. Nonetheless, the same cytokine patterns were observed in foam cells vs. macrophages with a statistically significant increase in the secretion of TNF-α, IL-1β, CXCL8, and decreased expression of IL-10, IL-6, and CXCL9 in foam cells compared to macrophages.

The ratio between inflammatory cytokines and the anti-inflammatory IL-10 is informative in monocytes and macrophages. This simple metric illustrated the difference between foam cells and macrophages in terms of *Mtb* and LPS responses (Figure 4B). The ratio of inflammatory IL-1β, TNF-α, and IL-6 vs. anti-inflammatory IL-10 was significantly higher in response to *Mtb* vs. LPS and was increased in foam cells compared to macrophages.

To determine whether the increase in cytokines observed in *Mtb*-infected foam cells was related to NF-κB-mediated inflammation, we used the THP1-Lucia^TM^ reporter cell line (Stedman et al., 2018) to monitor NF-κB activation in response to *Mtb* infection or LPS exposure. In this experiment, foam cells induced by oleic acid, linoleic acid or a mixture of both were used. A significant increase in reporter activity was observed in all *Mtb*-infected cells compared to uninfected controls (Figure 4C). Linoleic-acid-derived foam cells showed the highest activation of NF-κB, followed by foam cells induced by oleic acid alone, and the mixture of the two fatty acids. Macrophages showed the lowest activation of NF-κB.

TNF-α is a NF-κB-dependent cytokine (Domingo-Gonzalez et al., 2016). To confirm that the NF-κB activation observed by the luciferase reporter led to the production of TNF-α, we measured the level of this cytokine in the same culture supernatant of *Mtb*-infected cells and uninfected controls. As shown in Figure 4D and Supplementary Figure S8, TNF-α production indeed correlated with NF-κB activation.

## Discussion

The phagocyte system in *Mtb* disease is highly heterogeneous and in addition to macrophages, the cells of the family adopt specialized phenotypes recognized as epithelioid cells, foam cell macrophages and multinucleated Langhans giant cells. In this study, we set out to assess the phagocytic ability of macrophages and fatty-acid-induced foam cells, using primary monocyte-derived macrophages as well as macrophages from THP-1 reporter cell lines.

We began with a detailed characterization of the fatty acid composition of triglycerides and phospholipids in macrophages and foam cells. By microscopy, we observed that in all macrophage cultures, there is a background level of foam cell formation detectable by staining of neutral lipids. These foam cells appear in human-serum-rich cultures; serum de-lipidation dramatically decreases their formation (not shown). When we supplemented the culture with fatty acids, the foam cell phenotype increases and all cells eventually show signs of lipid droplet accumulation. The droplets seem to be of a consistent size and do not coalesce.

Interestingly, our lipid analysis shows that despite microscopic evidence of lipid droplets, the control cultures did not accumulate TGs at all. Only cells exposed to the fatty acids oleic and linoleic acid accumulated triglycerides. The profound TG accumulation observed upon fatty acid supplementation is presumably mediated via the action of the diacylglycerol O-acyltransferase, DGAT-1 (Guerrini et al., 2018; Jaisinghani et al., 2018; Greenwood et al., 2019). This is in accordance with other reports, where macrophages loaded with oleic acid (den Hartigh et al., 2010; Sarathy et al., 2016; Greenwood et al., 2019), linoleic acid or a mixture of both led to the accumulation of TG-rich lipid droplets.

We further showed that the lipid deposits could be largely tailored according to the composition of the fatty acid supplement. While the effects of fatty acid supplementation on the phospholipid content of the cells were less pronounced, the proportions of oleic acid, linoleic acid or both in the total phospholipid pool were enriched with the corresponding fatty acid/s provided in the medium at the expense of palmitic, stearic and arachidonic acid, the proportions of which were reduced.

Fatty acids are known to influence cell membrane fluidity, adhesion, phagocytosis, signaling and other cellular processes (Wymann and Schneiter, 2008; Papackova and Cahova, 2015; Schumann, 2016; de Carvalho and Caramujo, 2018). The changes upon fatty acid exposure are so profound and varied, their repercussions are likely to be complex, affecting multiple cellular pathways/processes. At the level of the membrane, the changes in composition of the TGs and phospholipids could affect fluidity and, for example, the ability of cells to form lipid rafts. TG and PL compositional changes could also affect organelles such as lysosomes, phagosomes, peroxisomes and mitochondria – all important for the immune response. Furthermore, the fatty acids and TGs could impact upon the function of GPCRs, nuclear receptors and signaling cascades. The droplets themselves could have a hindering effect due to the fact that they occupy a large part of the macrophage cytoplasm. Clearly, further investigation will be required in order to dissect the contributions of the different pathways in these models.

Foam cells formed by fatty acid exposure are fragile and in tissue culture plastic, they can be observed but not detached for further studies. In this study, we were able to preserve the granularity and obtain a detailed FACS profile of foam cells by culturing cells in non-adherent Lumox hydrophobic dishes. This surface does not interfere with the phagocytic process and enables easy cell detachment. Fixing the bacteria enabled us to focus on the macrophage phagocytic ability without the complicating element of active invasion by live *Mtb*. Furthermore, fixing the bacteria instead of the cells enabled us to avoid alterations to the cells’ lipid droplet profiles. Our results showed heterogeneity in the foam cells generated under the conditions tested, with large, intermediate and small macrophages, both lipid laden or not, being observed. These differences have not been described by FACS analysis with this degree of clarity previously. We have no clear hypothesis for this cell heterogeneity which is an intriguing finding that warrants further investigation. However, as our experimental model is based on fatty acid exposure, this observation may be due to differences in fatty acid transport or TG synthesis. Furthermore, our results showed a decrease in the phagocytosis of fixed, FITC-stained *Mtb* by foam cells compared with macrophages. This finding was corroborated in monocyte-derived macrophages and THP-1-derived macrophages.

However, the most striking difference that we observed was in the number of dead cells elicited by *Mtb* exposure in macrophages vs. foam cells, which were largely protected and survived the challenge. Macrophage death is one of the recognized mechanisms by which *Mtb* evades the host immune system. The foam cell survival vs. death by necrosis, apoptosis, and pyroptosis – a spectrum of which has been observed following macrophage infection with virulent *Mtb* (Divangahi et al., 2013) – deserves further scrutiny. However, the heterogeneity in foam cell formation, phagocytosis and asynchrony in infection, coupled with the complexity of cell death modalities, pose a challenge at the cell population level, and more sophisticated sorting strategies will be required to elucidate precise inhibitory mechanisms at the single-cell level.

In addition, foam cells derived from either human or THP-1 macrophages were moderately restrictive for *Mtb* growth, as evidenced by CFU enumeration and confirmed by fluorescence-based readout. *Mtb* growth arrest has been observed in some foam cell models (Peyron et al., 2008; Daniel et al., 2011), but not in others (Genoula et al., 2018; Jaisinghani et al., 2018; Knight et al., 2018). In earlier studies, foam cells were associated with upregulation of *Mtb* genes in the DosR-regulated “dormancy” regulon and accumulation of intracytoplasmic lipid inclusions (ILI) in *Mtb* (Peyron et al., 2008; Daniel et al., 2011; Kapoor et al., 2013). Induction of the regulon controlled by the DosRST two-component system is a hallmark feature of *Mtb* that has entered into a state of non-replicating persistence in response to the cellular stressors of hypoxia, NO and CO (Park et al., 2003; Voskuil et al., 2003; Kumar et al., 2007, 2008). The DosR regulon includes the triacylglycerol synthase-encoding *tgs1* gene, which is responsible for TG accumulation in *Mtb* under these conditions (Sirakova et al., 2006), and thus provides the link between non-replicating persistence and ILI accumulation in *Mtb*. Together with the evidence associating non-replicating persistence with phenotypic drug tolerance (Gold and Nathan, 2017), this link has underpinned the notion that ILI-containing bacilli, such as those detected in the sputum of TB patients, are “fat and lazy” (Garton et al., 2008) and phenotypically drug tolerant (Turapov et al., 2016). However, in contrast to these studies, *Mtb* showed no significant growth impairment in necrosis-associated foamy macrophages (NFAMs) (Jaisinghani et al., 2018) and showed even better growth in human macrophages which became foam cells by incubation with oxidized low-density lipoprotein than in resting macrophages (Vrieling et al., 2019), further highlighting differences between the various models.

In our study, *Mtb* was able to grow in foam cells, albeit more slowly than in resting macrophages. Interestingly, however, the growth attenuation observed in foam cells did not impact the efficacy against intracellular Mtb of any of the anti-TB drugs tested, all of which showed equivalent bactericidal activity against *Mtb* in foam cells vs. resting THP-1-derived macrophages when the drug was applied to infected cells at a concentration 10-fold above the *in vitro* MIC_90_. Within macrophages, *Mtb* encounters an environment that differs substantially from broth culture and is subject to host-dependent pharmacokinetic phenomena which may modulate drug activity and availability (see, for example, Dhillon and Mitchison, 1989; Hartkoorn et al., 2007; Sorrentino et al., 2016). Furthermore, the intracellular concentration of TB drugs can be influenced by the action of drug exporters expressed by macrophages, as shown by Hartkoorn et al. (2007) who found that rifamipicin and ethambutol are substrates for P-glycoprotein. Sorrentino et al. (2016) compared the MIC_90_ values for rifampicin, isoniazid and moxifloxacin in THP-1 macrophages vs. *in vitro* culture, and found that MIC_90_ of rifampicin was the same in both assays, whereas the MIC_90_ values for isoniazid and moxifloxacin were 4-fold and 9-fold higher, respectively, in macrophages vs. *in vitro*. By using a drug concentration 10-fold higher than the *in vitro* MIC_90_ – and hence, above the MIC_90_ in THP-1 cells, albeit to differing extents for the four drugs – we ensured that the sensitivity of the assays comparing drug efficacy in resting macrophages vs. foam cells would not be limited by use of sub-inhibitory drug concentrations. If the slower growth of *Mtb* observed in foam cells was indeed indicative of drug “tolerance,” as defined by Brauner et al. (2016), this would be expected to lead to a difference in time-kill kinetics, as reflected by an increase in the “minimum duration of kill” (MDK; Brauner et al., 2016) in foam cells vs. resting macrophages. The fact that no significant differences in the time-kill kinetics were observed for any of the drugs tested in the experiment shown in Figure 3D suggests that the growth attenuation – and any potential drug tolerance arising from this – was not sufficient to result in an observable shift in the MDK. The question of whether foam cells influence drug accumulation has been addressed for some TB drugs by other groups. Specifically, Blanc et al. (2018) showed that fluoroquinolone uptake was higher in foam cells than resting macrophages. Greenwood et al. (2019) showed that bedaquiline (BDQ) accumulates in lipid droplets and selectively targets *Mtb* in foam cells. The concentration of bedaquiline used by Greenwood et al. was 2.5 mg/L, which is comparable to the concentration used in our study (3.1 mg/L). These observations notwithstanding, we did not observe significant differences in the time-kill kinetics of either moxifloxacin or bedaquiline in our foam cell model. The unaltered efficacy of bedaquiline under the assay conditions tested in our study contrasts the findings of Greenwood et al. (2019) who found that this drug was more efficacious in *Mtb*-infected human macrophages in which lipid droplets were induced prior to drug treatment than in macrophages pharmacologically depleted of lipid droplets. These authors concluded that bedaquiline selectively targets *Mtb* in foam cells and proposed that lipophilic drugs accumulate in lipid droplets and are transferred to the bacilli as the lipid droplets are consumed. The reasons underlying these discrepant findings are presently unclear.

The initial response of macrophages to *Mtb* involves a variety of cytokines and chemokines which recruit granulocytes, and further inflammatory monocytes and macrophages to the nascent granuloma. We show that foam cells have increased proinflammatory potential with exacerbation of the acute phase cytokines IL-1β and TNF-α as well as upregulation of chemokines conventionally associated with the attraction of phagocytes and Th1 cells. Our results are consistent with those of Jaisinghani *et al.* who showed that Mtb-infected NFAMs release 1.5–3-fold greater release of TNF-α, IL-1β, IL-1α, IL-6, GCSF, and GMCSF compared to *Mtb*-infected THP-1 macrophages (Jaisinghani et al., 2018). CCL2 exhibits chemotactic activity for monocytes and basophils, CCL5 is chemotactic for T cells, eosinophils and basophils, and CXCL10 recruits monocytes, T cells, NK cells and dendritic cells to the target cells. The increase in inflammatory cytokines was concomitant with a decrease in the production of IL-10. However, as cytokine and chemokine panels are biased and do not include ligands for cells of the Th2 arm of the immune response, the possibility that there is a general dysregulation of cytokine production in foam cells cannot be excluded.

Mycobacterium is a complex bacterium the recognition of which involves opsonic and non-opsonic receptors, as well as numerous other components of the phagocytic synapse. It is well known that *Mtb* recognition at the level of the cell membrane also involves the inflammation-related pattern recognition receptors, TLR4 and TLR2. LPS stimulation is a simpler model that leads to activation of NF-κB and IRF pathways in macrophages. We investigated whether foam cell formation could also increase the inflammatory cytokine and chemokine response to LPS and found indeed that foam cells produced more inflammatory cytokines than macrophages when exposed to LPS, in a similar fashion to *Mtb*-infected cells.

At the stimulus level, purified LPS is in general a stronger stimulus than *Mtb* and induced higher production of cytokines and chemokines with one exception – CXCL8. CXCL8 is the main granulocyte chemoattractant and has been shown to be elevated in supernatants of macrophages and in bronchoalveolar lavage from patients with pulmonary TB (Zhang et al., 1995). The *Mtb* cell wall components, lipoarabinomannan (LAM), lipomannan (LM), and phosphoinositolmannoside (PIM) have been linked to CXCL8 release, and neutralizing antibodies to TNF-α and IL-1β, alone or in combination, shown to abrogate CXCL8 production (Zhang et al., 1995). In our study, CXCL8 was not only consistently higher in foam cells vs. macrophages in a basal state, but further exacerbated by *Mtb* and not LPS. Differences in the promoter of CXCL8 vs. the inflammatory cytokines would merit further investigation.

Finally, looking at cytokine levels alone misses an important feature, namely, the ratio between inflammatory cytokines and the anti-inflammatory cytokine, IL-10. For example, a high ratio of TNF-α/IL10 or IL-1β/IL-10 has been used within the M1/M2 macrophage activation paradigm to indicate M1 or classical activation. In our model, the ratio between the two inflammatory cytokines vs. IL-10 was higher for *Mtb* infection than purified LPS exposure and, as expected, increased in foam cells. The activation of the nuclear factor NF-κB was also higher in foam cells than macrophages and correlated with TNF-α production in the same cells. Based on our findings, we therefore conclude that foam cells have increased capacity to recruit neutrophils, phagocytes and Th1 cells to early granulomata and contribute to the exacerbated inflammatory response.

Whilst this is the first observation about cell death resistance by foam cells, our results add to a growing body of work on foam cell formation and function in various host species and models, using different mycobacteria (Supplementary Table S1). The key findings from the published literature, as summarized in Supplementary Table S1, not only highlight the diversity of experimental models and approaches, but also underscore the lack of consensus regarding the inflammatory potential of the cells, as determined from different studies. The disparate and often conflicting conclusions reached suggest that the foam cell field has reached a level of complexity that now requires a nomenclature and culture guideline to enable better contextualization of new experimental data. Greater specificity is needed when using models and reporting culture standards. Furthermore, lipid regulatory mechanisms warrant further research. The studies by Almeida et al. (2014) and Knight et al. (2018) demonstrated that lipid droplets formation is not essential for bacterial nutrition or growth; but it is a host directed response required for the synthesis of host protective eicosanoids in order to control the infection. Increased cytokine levels can have paracrine effects on bystander macrophages and cells by increasing their inflammatory profile. Notably, Jaisinghani et al. (2018) showed that *Mtb*-induced necrosis stimulates TG accumulation in foam cells as a bystander response. Similarly, Greenwood et al. (2019) showed that *Mtb* exposure induced lipid droplet accumulation in uninfected bystander cells. Once such TG-loaded cells encounter bacilli, they mount a higher pro-inflammatory response as evidenced by elevated levels of TNF-α, IL-1β, IL-1α, IL-6, G-CSF, and GM-CSF (Jaisinghani et al., 2018). However, an outstanding question not addressed in this study or ours, albeit worthy of future investigation, is whether the cytokines produced by foam cells could increase foam cell formation in the presence of lipids.

Our findings suggest potential pathways for nutritional intervention and modulation of the response to *Mtb* infection, which we could apply in future exploiting lipid supplementation and established lipid related inhibitors, in more advanced clinical studies. The role of different dietary lipids needs further elucidation, but our results suggest that we could exploit the decreased phagocytic capacity of foam cells, and increased survival and inflammatory potential against *Mtb*, to alter the outcome of infection in patients and reduce transmission. The potential of fatty acid supplementation to modulate anti-TB responses is therefore an appealing prospect that warrants further investigation.

## Data Availability Statement

The raw data supporting the conclusions of this article will be made available by the authors, without undue reservation, to any qualified researcher.

## Ethics Statement

The studies involving human participants were reviewed and approved by the small fresh blood donations of 50 ml or less were acquired in the laboratory under ethics approval (UEC/2017/052/FHMS), and leukocyte cones were acquired from NHSBT blood services under ethics permit REC (17/LO/1877). The patients/participants provided their written informed consent to participate in this study.

## Author Contributions

PA, SG, VM, and FM conceived and designed the experiments. PA, TC, FM, and BF performed the experiments. PA, BF, SG, VM, and FM analyzed the data. PA, SG, VM, and FM wrote the manuscript. All authors contributed to the article and approved the submitted version.

## Conflict of Interest

The authors declare that the research was conducted in the absence of any commercial or financial relationships that could be construed as a potential conflict of interest.
