# Corrigendum: Foam Cells Control *Mycobacterium tuberculosis* Infection

**DOI:** 10.3389/fmicb.2020.594142

**Published:** 2020-10-27

**Authors:** Pooja Agarwal, Theo W. Combes, Fariba Shojaee-Moradie, Barbara Fielding, Siamon Gordon, Valerie Mizrahi, Fernando O. Martinez

**Affiliations:** ^1^South African Medical Research Council/National Health Laboratory Service/University of Cape Town, Molecular Mycobacteriology Research Unit, Division of Medical Microbiology, Department of Pathology, Department of Science and Innovation/National Research Foundation, Centre of Excellence for Biomedical TB Research and Wellcome Centre for Infectious Diseases Research in Africa, Institute of Infectious Disease and Molecular Medicine, University of Cape Town, Cape Town, South Africa; ^2^Faculty of Health and Medical Sciences, University of Surrey, Guildford, United Kingdom; ^3^Graduate Institute of Biomedical Sciences, College of Medicine, Chang Gung University, Taoyuan City, Taiwan; ^4^Sir William Dunn School of Pathology, University of Oxford, Oxford, United Kingdom

**Keywords:** tuberculosis, foam cells, macrophage, lipid droplets, cytokines, inflammation

In the original article, there was a mistake in Figure 1, **Panel A** as published. Some images in Panel A were mistakenly duplicated. The images for Linoleic acid, D1, 200 μM and 400 μM were repeat copies of the images in D1, 200 μM and 400 μM for Linoleic acid + Oleic acid. The corrected Figure 1, **Panel A** appears below.

**Figure 1:**
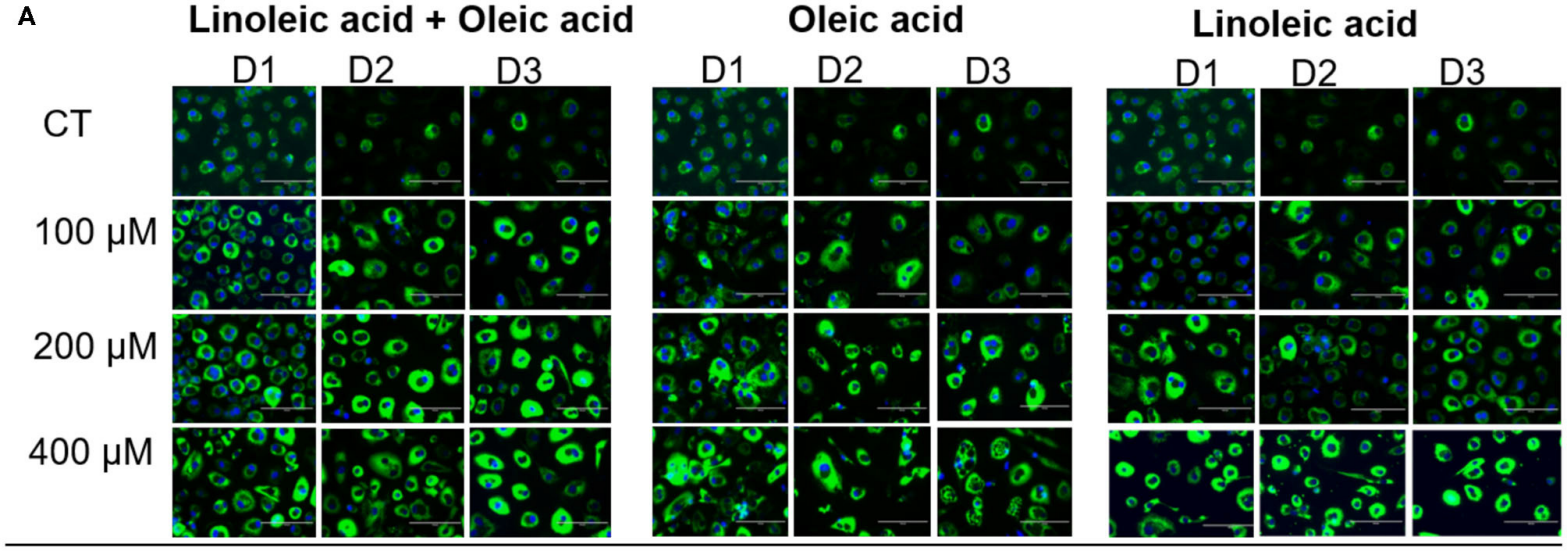


The authors apologize for this error and state that this does not change the scientific conclusions of the article in any way. The original article has been updated.

