# Supplementary material for: Foam Cells Control Mycobacterium tuberculosis Infection
Source: Front Microbiol. 2020 Jul 9;11:1394. doi: 10.3389/fmicb.2020.01394 (PMC7381311; doi:10.3389/fmicb.2020.01394)
Supplement: Supplementary file 1 [file Data_Sheet_1.docx]

**SUPPLEMENTARY INFORMATION**

**Foam Cells Control *Mycobacterium tuberculosis* Infection**

**Pooja Agarwal^1^, Theo Combes^2^, Fariba Shojaee-Moradie^2^, Barbara Fielding^2^, Siamon Gordon^3,4^, Valerie Mizrahi^1*^, Fernando O. Martinez^2*^**

**Figure S1. Viability assessment of foam cell populations produced by exposure to oleic acid.** Foam cell viability was assessed using a Live/Dead-viability/cytotoxicity kit (Invitrogen) based on Calcein-AM and EthD-1 staining, as per the manufacturer’s instructions. Stained cells were observed under a ZOE™ Fluorescent Cell Imager (Bio-Rad). Live cells convert nonfluorescent cell-permeant calcein AM to the intensely green fluorescent calcein by the enzymatic action of esterase while EthD-1 enters dead cells with a compromised cell membrane and binds to nucleic acid producing a bright red fluorescence signal.

**
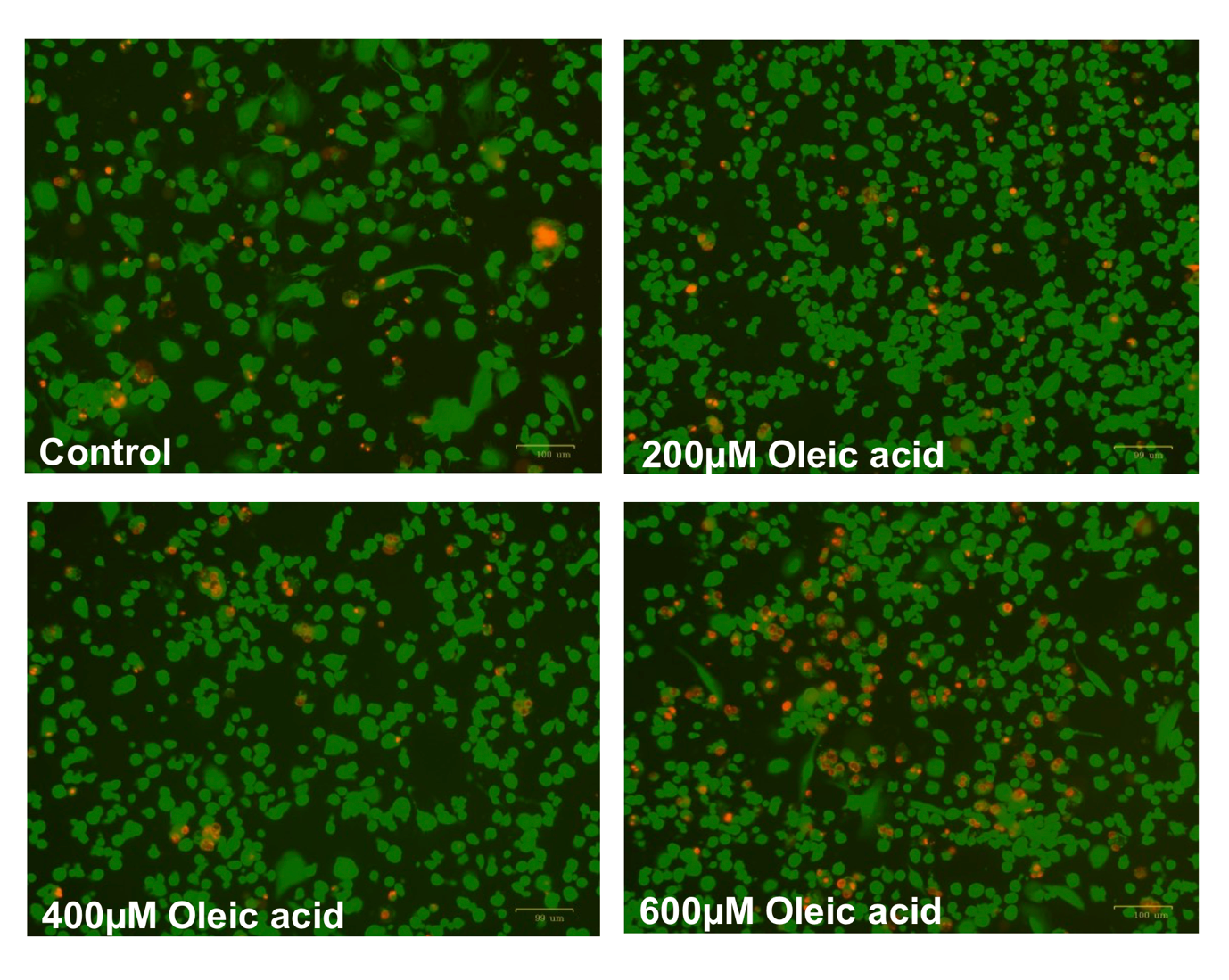
**

**Figure S2. Foam cells accumulate lipid droplets in an oleic acid dose dependent manner.** THP-1 macrophages were differentiated into foam cells by exposing them to oleic acid at the indicated concentrations for 24 h or left untreated to serve as resting macrophages. Thereafter, foam cells and resting macrophages (mock-treated and processed in parallel) were fixed with 4% paraformaldehyde and stained with Nile Red (10µg/ml, Sigma) to detect lipid droplets. Stained cells were imaged by ZEISS AXIO SCOPE.A1 microscope at 100× magnification. Lipid droplets are visible as bright spots in the cells. Scale bar is 10 µm.

**
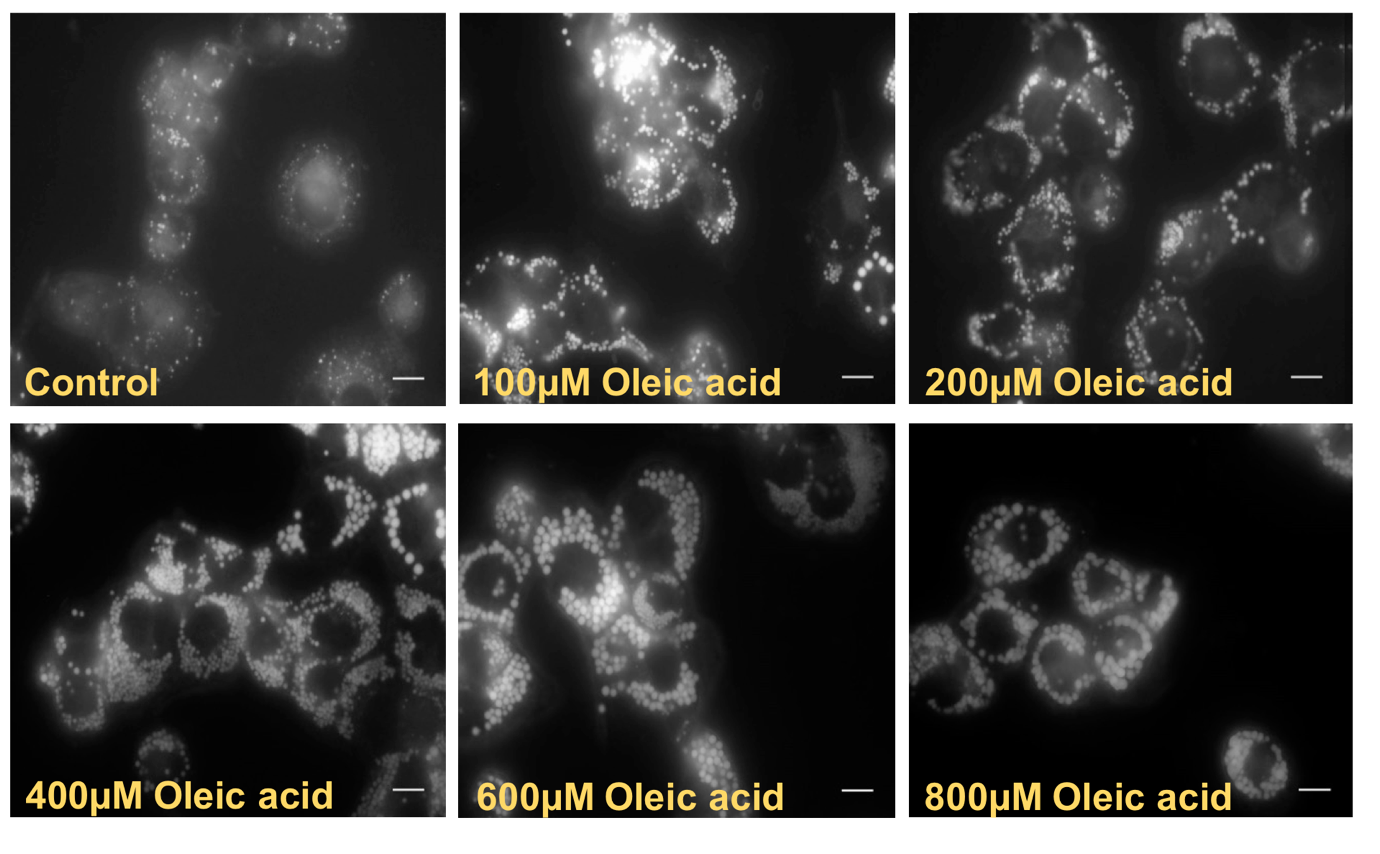
**

**Figure S3. Durability of lipid droplets in foam cells maintained in the absence of exogenous lipid.** THP-1-derived foam cells generated by exposure to 400µM oleic acid were maintained in oleic-acid-free cell culture medium for 6 days. Each day, lipid droplets were stained with Nile Red and observed using a ZEISS AXIO SCOPE.A1 microscope at 100 × magnification. Lipid droplets are maintained over 6 days although there was a noticeable reduction in droplet size over time. Scale bar = 10µm.


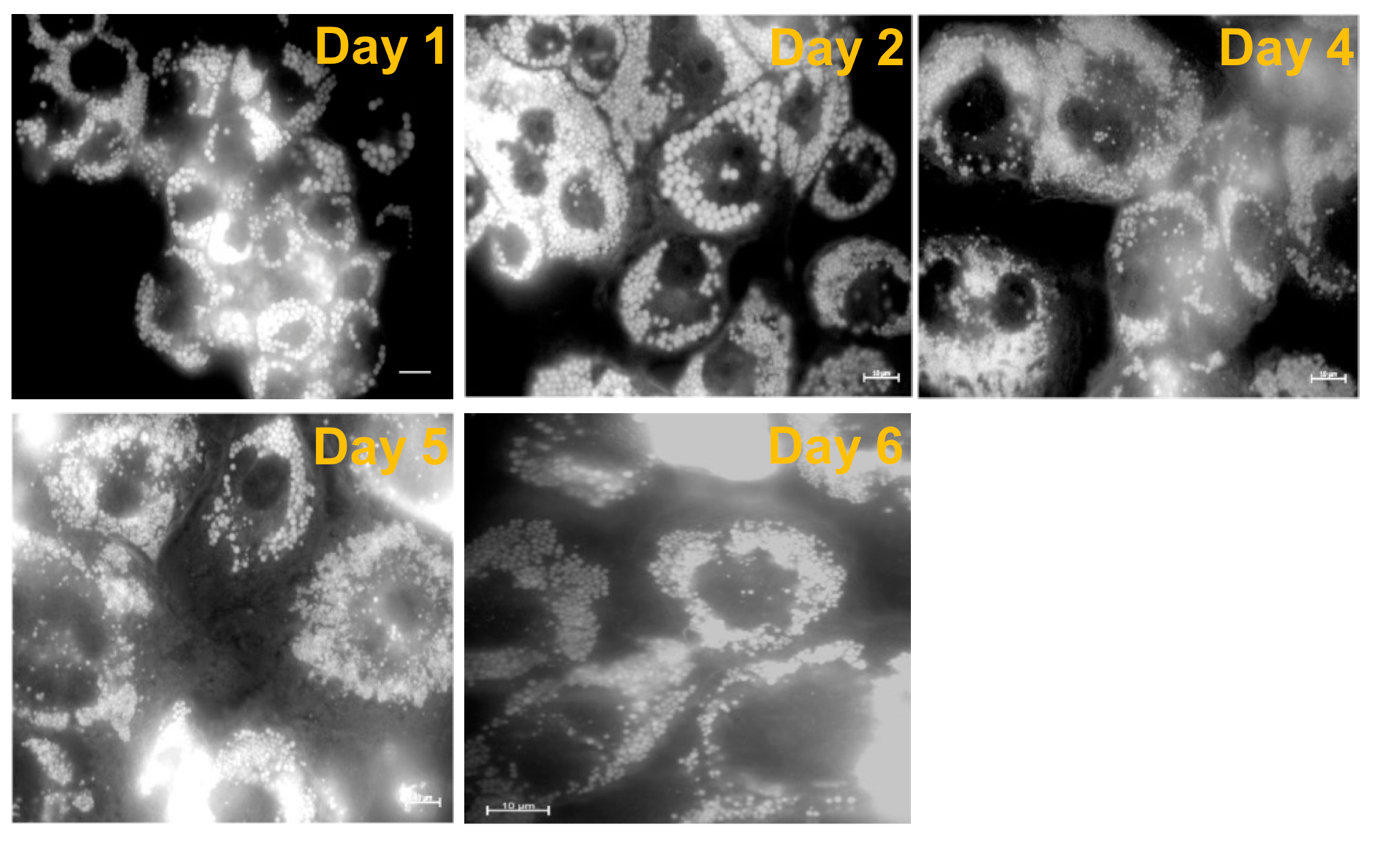


**Figure S4. *Mtb* shows retarded growth in foam cells.** THP-1-derived foam cells and resting macrophages were infected with *Mtb*-H37Rv::mCherry at an MOI of 5, as described in Materials and Methods. (**A**) Foam cells and resting cells were imaged at the indicated time points using a ZOE™ Fluorescent Cell Imager (Bio-Rad) under bright field while bacteria were imaged in the red channel. Shown in figures are the merged images taken in both the channels, *Mtb-*H37Rv::mCherry in red and foam cells and resting cells as unstained. (**B**) Fluorescence on day 1, 3, 5 and 7 was measured using a Fluostar plate reader (BMG Biotech) (excitation, 544 nm; emission, 590 nm). Resting macrophages, red squares; foam cells, black circles. The data represent the mean ± SD from triplicate wells (n=2). Statistical analysis was performed using the Bonferroni-Sidak method, ***p*=0.00451, ***p*=0.00684 for day 5 and day 7, respectively. RFU, relative fluorescence units.


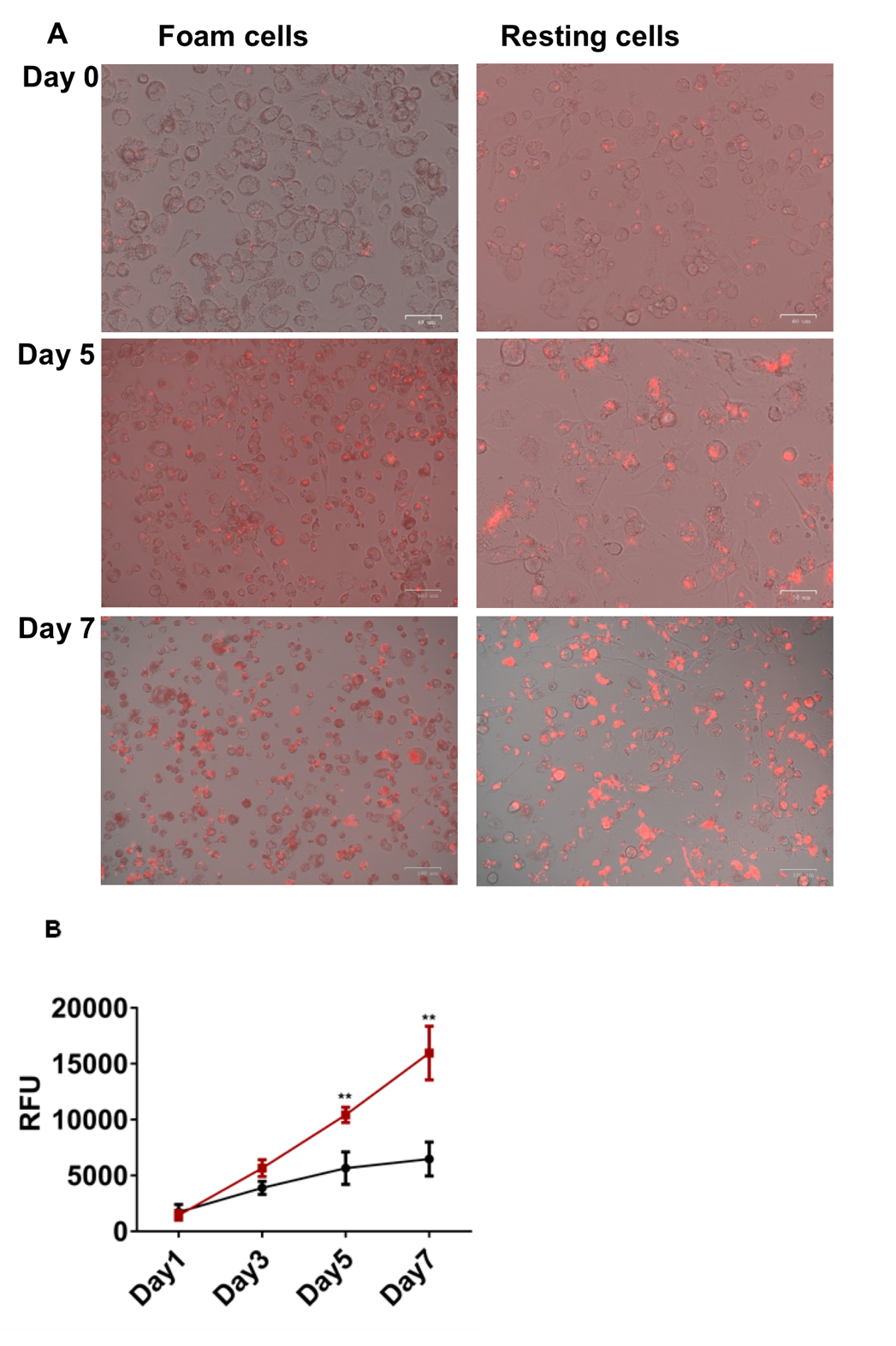


**Figure S5. Supplement to Figure 2.** A) Quantification of the percentage of FITC positive cells regardless of size or granularity shows a decrease in phagocytosis at 24 hours. The pValue for this comparison was 0.07. B) Percentage of dead cells corresponding to Figure 2D.

**
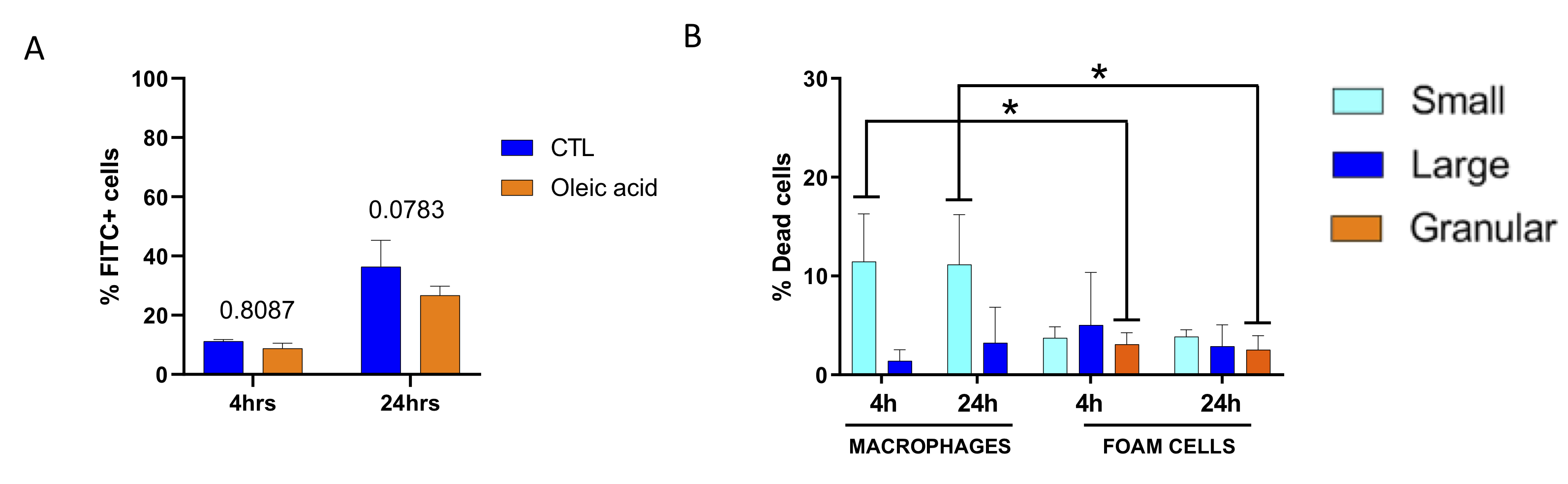
**

**Figure S6. TB-drugs are not toxic to cells.** THP-1 -derived foam cells and resting cells were exposed to drugs as described in methods. Toxicity of drugs was measured by alamar blue assay on day 1, 2, 4, and 6 using a Fluostar plate reader (BMG Biotech) (excitation, 560 nm; emission, 590 nm). Data is represented as average ±SD of three different experiment.


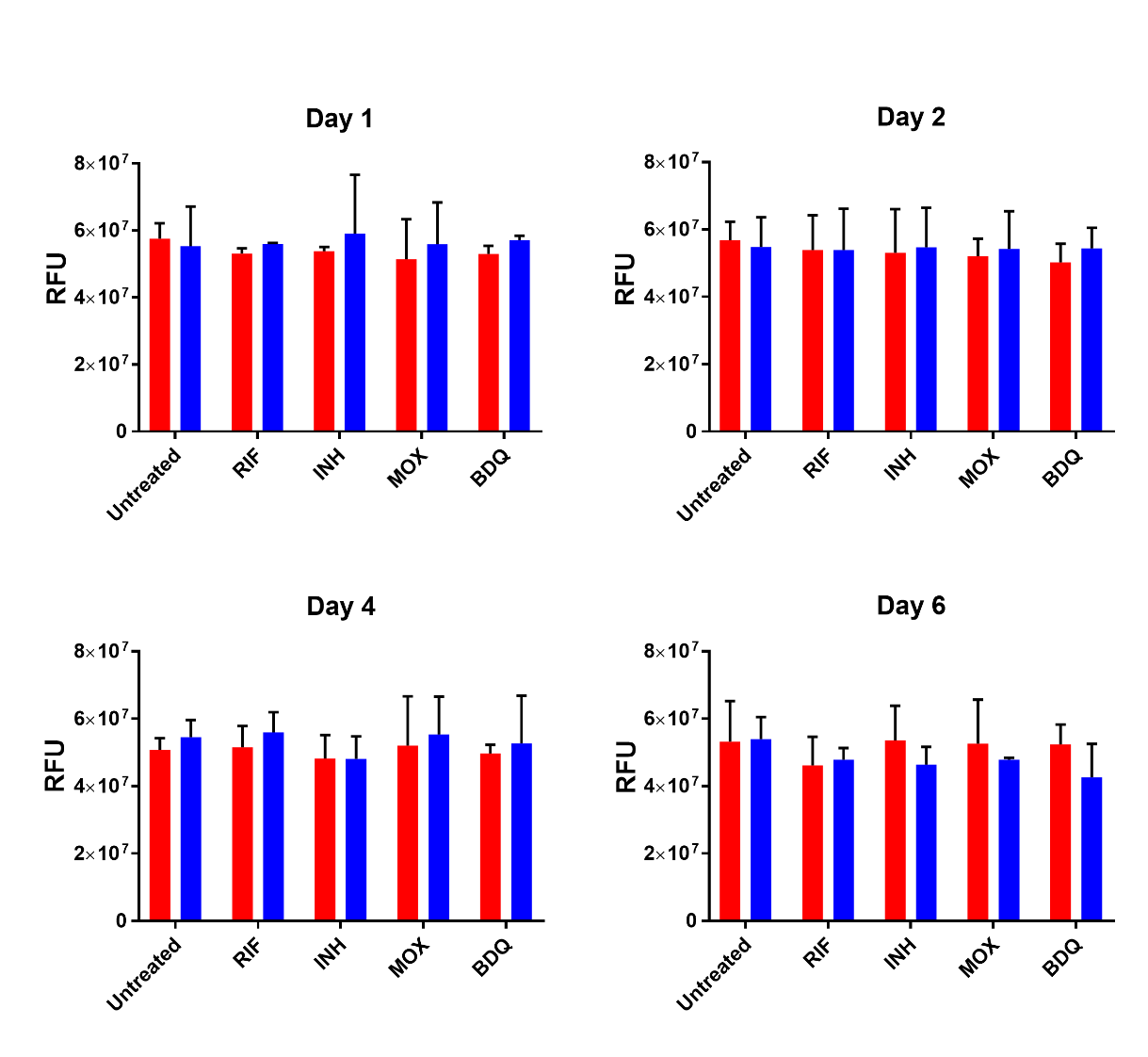


**Figure S7. Comparative chemokine secretion by oleic-acid-induced THP-1 foam cells versus resting macrophages infected with *Mtb* or exposed to LPS**. Culture supernatants from *Mtb*-infected, LPS-exposed or uninfected/unexposed control cells (None) were collected 24 h and 48 h after *Mtb* infection or LPS exposure and analysed by flow cytometry using a cytometric bead array kit as described in Materials and Methods. Statistical differences were assessed with two- way ANOVA, Sidak’s was used as multiple comparisons test. The ANOVA showed *p*-values for both treatment and dose <0.0001. Statistically significant differences between foam cell and macrophages for each stimulus are indicated with an asterisk; **p*<0.05, ***p*<0.01, ****p*<0.001, *****p*<0.0001; ns, not significant.

#
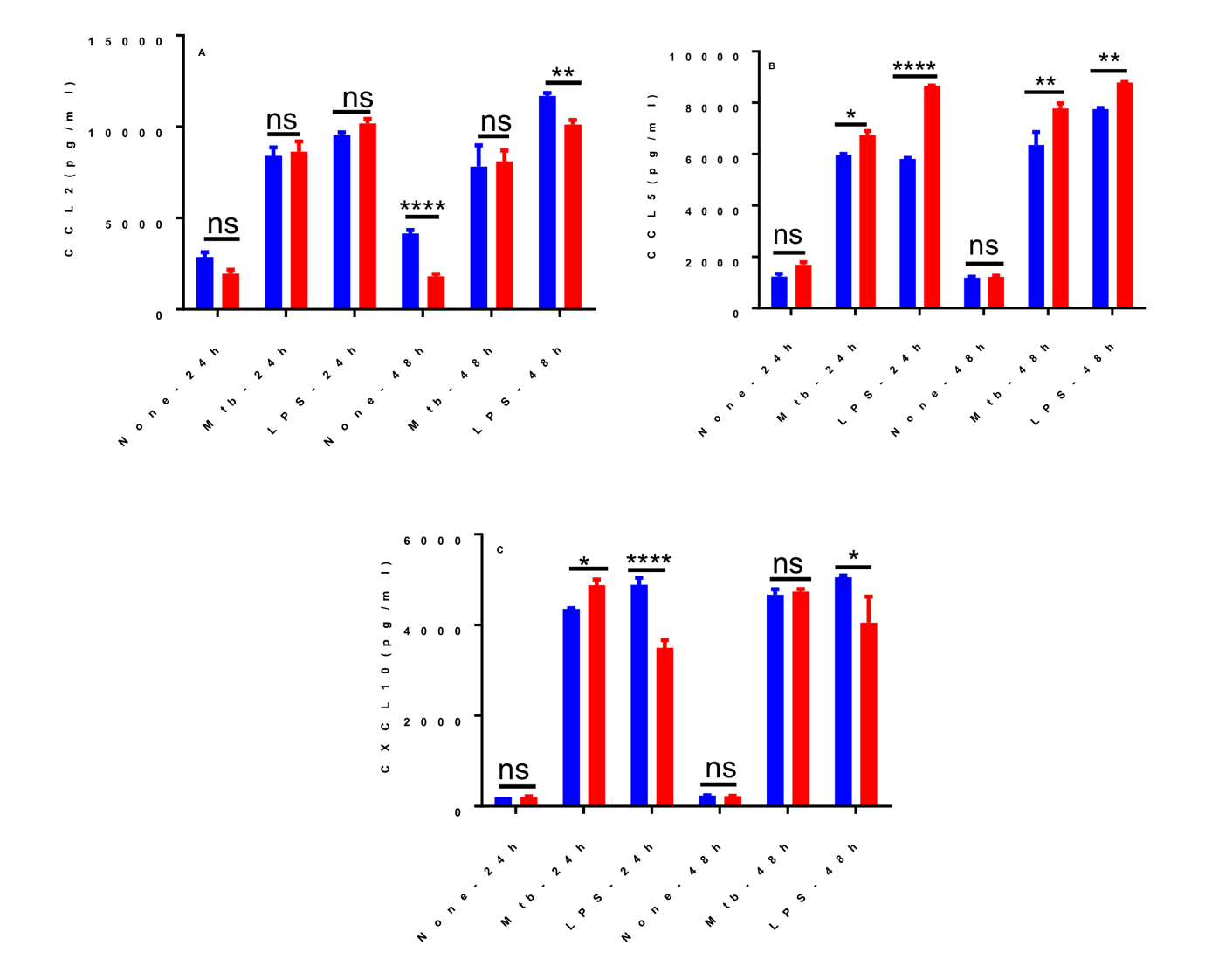


**Figure S8.** **Control values for Figure 4D.** TNF-α secretion in culture supernatant was measured by ELISA and the data were correlated to reporter luminescence. Black Dots close to the start points are the control group of all Mtb-infected foam cells model. Data are represented as average ± SD from a representative experiment (n = 6), **p*<0.05, ***p*<0.01, ****p* <0.001, *****p* <0.0001 by one-way ANOVA with Bonferroni post-tests.

**Table S1. Key findings from studies investigating the association between foam cells and infection with *M. tuberculosis* (Mtb) or other mycobacteria**

| **Reference** | **Host cell** | **Mycobacterial species** | **Infection model and key findings** | **Mycobacterial characteristics** | | | |
| --- | --- | --- | --- | --- | --- | --- | --- |
|  |  |  |  | **Growth, physiology and metabolism** | **Anti-TB drug susceptibility** | **Accumulation of intracytoplasmic lipid inclusions (ILI)** | **Detected as Acid Fast Bacilli (AFB) or by Nile Red (NR) staining** |
| (Peyron et al., 2008) | Human PBMC | *M. smegmatis* Mtb | PBMC were infected with either *M. smegmatis* or Mtb for 3 days or 11 days to produce *in vitro* granulomas in which foam cell formation was observed   - Mtb but not *M. smegmatis* induces the formation of foam cells - Oxygenated mycolic acid induces foam cell formation - Phagocytic and bactericidal activities are arrested in foam cells | - Mtb persists in a non-replicating state in foam cells - “Dormancy” genes upregulated | ND | - ILI observed in Mtb residing in foam cells | - AFB+ Mtb found in foam cells from lymph node biopsies of TB patients |
| (Mattos et al., 2010) | Human & murine macrophages | *M. leprae* | *M. leprae* induces lipid droplet formation in human and murine macrophage as observed after 48 h   - *M. leprae* induces the secretion of soluble factors in bacterium-associated cells which act in a paracrine-signalling circuit to induce lipid droplet formation in uninfected cells - TLR2, TLR6, and cytoskeleton are involved in *M. leprae* induction of lipid droplet formation | ND | ND | ND | ND |
| (Mattos et al., 2011) | Human Schwann cells | *M. leprae* | Human Schwann cells were infected with *M. leprae*, LD formation was observed after 48 h   - TLR2 signalling is not essential for *M. leprae* -induced LD formation in SCs - LD recruits to pathogen containing phagosome | - Inhibition of lipid droplet recruitment to phagosome decreases *M. leprae* survival in Schwann cells | ND | ND | ND |
| (Daniel et al., 2011) | Human macrophages  & THP-1 cells | Mtb H37Rv  *M. smegmatis* | Cells were infected first before incubation under 1% or 21% oxygen   - Macrophages accumulate lipid droplets in 1% oxygen which increases in infected cells - Mtb-infected macrophages show slightly higher TAG accumulation than *M. smegmatis*-infected macrophages | - Mtb replication in hypoxic host cells severely curtailed in contrast to normoxic cells in which Mtb grows over time - DosR-associated Mtb genes upregulated under hypoxia | - Intracellular Mtb showed phenotypic tolerance to isoniazid and rifampicin under hypoxia but not under normoxia | - Mtb accumulates ILI in foam cells | AFB-  NR+ |
| (Singh et al., 2012) | Human macrophages & THP-1 cells | Mtb H37Rv  Mtb H37Ra | Macrophages were exposed to H37Rv or H37Ra   - Macrophages accumulate lipid droplets only in response to H37Rv - Foam cells are not bactericidal | - Mtb grows | ND | ND | ND |
| (Kapoor et al., 2013) | Human PBMCs | Mtb H37Rv | - Infection of human PBMCs with Mtb resulted in the formation of *in vitro* granulomas - Foam cell formation not assessed | - DosR-associated genes upregulated | - Mtb shows phenotypic rifampicin resistance in an 8- day *in vitro* granuloma model | - Mtb accumulates ILI | AFB-  NR+ |
| (Podinovskaia et al., 2013) | Human macrophages & Murine macrophages | Mtb CDC1551 | Human macrophages infected with Mtb for 5 days then processed for EM  Murine macrophages exposed to 400 µM oleic acid for 24 h then infected with Mtb. Alternatively, macrophages were infected with Mtb for 5 days then exposed to 400uM oleate to study the trafficking of host-acquired lipids into intracellular Mtb   - Mtb infection induces foamy phenotype in human macrophages where Mtb is found associated with host lipid droplets. - Mtb infection leads to retention of the foamy macrophage phenotype while uninfected cells lose it | ND | ND | - Lipid was incorporated both into lipid droplets in the host cell and into ILI in Mtb | ND |
| (Lee et al., 2013) | Murine macrophages | Mtb Erdman  Mtb H37Rv- *icl1* mutant | Macrophages were incubated with 400 µM oleate alone or 400 µM oleate and 20 µCi of [1-^14^C] oleic acid, 20 µCi [1-^14^C] stearic acid, or  [1-^14^C] sodium propionate for 24 h then infected with Mtb | - Growth of the *icl1* mutant severely impaired in control (untreated) macrophages but rescued in macrophages preloaded with lipid droplets induced by oleate - Mtb in lipid-droplet-loaded macrophages incorporates host-derived fatty acids into PDIM | ND | ND | ND |
| (Almeida et al., 2014) | Mouse peritoneal macrophages | M*. bovis* BCG | Macrophages were infected with BCG then monitored for lipid droplet formation   - BCG infection induces lipid droplet formation in a TLR2-dependent manner with the cooperation of other factors | ND | ND | ND | ND |
| (Caire-Brandli et al., 2014) | Murine macrophages | *M. avium* | Macrophages were infected with *M. avium*, and at day 6 post-infection, cells were exposed to VLDL for 0-24 h, fixed and processed for EM   - Macrophages accumulate lipid droplets in a reversible manner. - Removal of VLDL leads to a rapid decline of lipid droplets | - *M. avium* cell division is arrested in VLDL-driven foam cells | ND | - *M. avium* accumulates ILI only in VLDL-exposed macrophages where it acquires lipid from the host - Removal of VLDL leads to a rapid decline in host lipid bodies, the concurrent disappearance of ILI from *M. avium*, and the immediate resumption of mycobacterial cell division | ND |
| (Holla et al., 2016) | Mouse peritoneal macrophages,  RAW 264.7 | *Mtb* H37Rv  *Mtb* MDR-JAL2287  H37Ra  *M. bovis* BCG  *M. smegmatis* | Macrophages were infected with mycobacteria, then lipid droplet accumulation monitored   - jumonji domain containing protein (JMJD) 3 is essential for foamy macrophage generation in a TLR2-dependent manner - *M. smegmatis* does not induce foamy phenotype in macrophages | ND | ND | ND | ND |
| (Barisch and Soldati, 2017) | *Dictyostelium*  *discoideum*-wild type  and DGAT1/ DGAT2 double mutant | *M. marinum* | *D. discoideum* either left untreated or incubated with 200 µM palmitic acid for 3 h before infection with *M. marinum.* | - *M. marinum* metabolically active and its growth not significantly different in wild type vs. mutant *D. discoideum* - Supplementation with fatty acid had no impact on mycobacterial growth | ND | - *M. marinum* accumulates ILI in wild type *D. discoideum* - In mutant *D. discoideum,* *M.marinum* accumulates ILI by using host-derived phospholipid | - NR+ in both wild type and mutant hosts |
| (Genoula et al., 2018) | Human  macrophage  Murine macrophage | Mtb H37Rv | Foam cell formation by exposure of cell-free pleural effusion from TB patients, Mtb lipid or Mtb   - Foam cells show immunosuppressive properties - IL-10 in pleural effusion is responsible for foam cell formation - TB pleural effusion-induced foamy macrophages secrete higher levels of IL-10 and lower levels of TNF-α upon stimulation with irradiated Mtb | - No difference in the uptake of Mtb by foam cells vs. control cells - Bacillary load increases over time in foam cells | ND | ND | ND |
| (Knight et al., 2018) | Human- macrophage  Murine macrophage | Mtb Erdman | IFNγ activated and un-activated macrophages were infected with Mtb   - Lipid droplets form only in activated macrophages - IFNγ activation or Mtb infection alone does not increase lipid droplet formation - Lipid droplets support host immunity - Lipid droplets are not an important source of nutrients for Mtb growth | - Macrophage lipid droplets are neither essential for Mtb growth nor required for cell intrinsic control of infection in this system | ND | - EM shows ILI accumulation by Mtb in un-activated macrophages, but not in IFNγ- activated macrophages | ND |
| (Jaisinghani et al., 2018) | THP-1 cells  Human macrophages | Mtb H37Rv | - Mtb infection of macrophages at MOI of 50 (bacteria per cell), but not at a MOI of 1 or 5, leads to foam cell formation, associated with >60% cell death by necrosis. - THP-1 macrophages lysed and lysate fed to macrophages for 8 days which led to lipid accumulation in necrosis-associated foamy macrophages (NAFM) - Mtb-infected NAFMs release 1.5- to 3-fold higher TNFα vs. normal, Mtb-infected THP1 macrophages - No difference in uptake of Mtb between NAFM and normal macrophages - IL-1β, IL-1α, IL-6, GCSF and GMCSF release upon Mtb infection is 2- to 2.5-fold higher from NAFMs vs. normal macrophages - TAG has an important role in the inflammatory response of NAFM to Mtb infection | - No significant difference in the growth of Mtb in normal macrophages and NAFM | ND | ND | ND |
| (Guerrini et al., 2018) | Human macrophages | Mtb H37Rv | Macrophages infected with Mtb and lipid droplet accumulation assessed   - Lipid droplet accumulation requires TNFα signalling - Uninfected (by-stander) cells also accumulate lipid droplets (albeit to a lesser extent) suggesting a role for TNFα secreted by infected cells in this effect | ND | ND | ND | ND |
| (Johansen et al., 2018) | Zebrafish embryos | *M. marinum* | Zebrafish embryos infected with *M. marinum*, then low density lipoprotein (LDL) content analysed in embryos   - LDL level increased in infected embryos - Depletion of LDL receptor decreased *M. marinum* burden - *M. marinum* infection-induced granuloma lipid accumulation reduced in LDL receptor-deficient embryos | *M. marinum* load increased in zebrafish embryos | ND | ND | ND |
| (Johansen et al., 2019) | RAW 264.7 murine macrophage cells | *M. avium subsp. paratuberculosis* (MAP) | Macrophages were infected with different strains of MAP   - Intracellular cholesterol levels significantly increased in macrophages infected with all MAP strains tested - MAP identified within cholesterol-rich areas of macrophages - MAP-infected macrophages showed strong upregulation of IL-1β, IL-6, MCP-1 & TNFα | ND | ND | ND | ND |
| (Vrieling et al., 2019) | Human macrophages | Mtb H37Rv | Macrophages incubated with oxidized LDL (oxLDL), acetylated LDL (acLDL) or LDL for 24 h to differentiate macrophages into foam cells prior to infection with Mtb   - oxLDL and acLDL but not LDL exposed macrophages accumulate lipid droplets - Phagocytic capacity unaffected by lipid treatment - oxLDL-treated macrophages produce significantly lower levels of TNFα and IL-6 after 24 h of infection. Other cytokines and chemokines are also lower in these cells vs. controls - acLD-treated macrophages produce intermediate levels of cytokine and chemokines vs. controls in response to Mtb infection - oxLDL supports Mtb intracellular survival through lysosomal cholesterol accumulation and by inhibiting Mtb localization to functional lysosomes in infected cells | Better growth of Mtb in oxLDL-induced foam cells compared to acLDL foam cells and untreated controls | ND | ND | ND |
| (Greenwood et al., 2019) | Human macrophages | Mtb H37Rv | Macrophages infected with Mtb   - Infected and bystander macrophages accumulate lipid droplets - Mtb found in close contact with lipid droplets - Mtb consumes lipid droplets over time   Macrophages treated with bedaquiline (BDQ) either before or after Mtb   - BDQ accumulates heterogeneously in Mtb within macrophages - Distribution of BDQ indistinguishable in Mtb irrespective of whether macrophages exposed to drug before or after infection - Lipid droplets accumulate BQD and serve a transferable reservoir of drug to Mtb - Inhibition of lipid droplet formation in macrophages reduces anti-TB efficacy of BDQ | Mtb grew over time in macrophages and consumed lipid droplets | Efficacy of BDQ increased in macrophages exposed to oleate | ND | ND |

ND, not determined

**REFERENCES**

Almeida, P.E., Roque, N.R., Magalhaes, K.G., Mattos, K.A., Teixeira, L., Maya-Monteiro, C., et al. (2014). Differential TLR2 downstream signaling regulates lipid metabolism and cytokine production triggered by Mycobacterium bovis BCG infection. *Biochim Biophys Acta* 1841(1)**,** 97-107. doi: 10.1016/j.bbalip.2013.10.008.

Barisch, C., and Soldati, T. (2017). Mycobacterium marinum Degrades Both Triacylglycerols and Phospholipids from Its Dictyostelium Host to Synthesise Its Own Triacylglycerols and Generate Lipid Inclusions. *PLoS Pathog* 13(1)**,** e1006095. doi: 10.1371/journal.ppat.1006095.

Caire-Brandli, I., Papadopoulos, A., Malaga, W., Marais, D., Canaan, S., Thilo, L., et al. (2014). Reversible lipid accumulation and associated division arrest of Mycobacterium avium in lipoprotein-induced foamy macrophages may resemble key events during latency and reactivation of tuberculosis. *Infect Immun* 82(2)**,** 476-490. doi: 10.1128/IAI.01196-13.

Daniel, J., Maamar, H., Deb, C., Sirakova, T.D., and Kolattukudy, P.E. (2011). Mycobacterium tuberculosis uses host triacylglycerol to accumulate lipid droplets and acquires a dormancy-like phenotype in lipid-loaded macrophages. *PLoS Pathog* 7(6)**,** e1002093. doi: 10.1371/journal.ppat.1002093.

Genoula, M., Marin Franco, J.L., Dupont, M., Kviatcovsky, D., Milillo, A., Schierloh, P., et al. (2018). Formation of Foamy Macrophages by Tuberculous Pleural Effusions Is Triggered by the Interleukin-10/Signal Transducer and Activator of Transcription 3 Axis through ACAT Upregulation. *Front Immunol* 9**,** 459. doi: 10.3389/fimmu.2018.00459.

Greenwood, D.J., Dos Santos, M.S., Huang, S., Russell, M.R.G., Collinson, L.M., MacRae, J.I., et al. (2019). Subcellular antibiotic visualization reveals a dynamic drug reservoir in infected macrophages. *Science* 364(6447)**,** 1279-1282. doi: 10.1126/science.aat9689.

Guerrini, V., Prideaux, B., Blanc, L., Bruiners, N., Arrigucci, R., Singh, S., et al. (2018). Storage lipid studies in tuberculosis reveal that foam cell biogenesis is disease-specific. *PLoS Pathog* 14(8)**,** e1007223. doi: 10.1371/journal.ppat.1007223.

Holla, S., Prakhar, P., Singh, V., Karnam, A., Mukherjee, T., Mahadik, K., et al. (2016). MUSASHI-Mediated Expression of JMJD3, a H3K27me3 Demethylase, Is Involved in Foamy Macrophage Generation during Mycobacterial Infection. *PLoS Pathog* 12(8)**,** e1005814. doi: 10.1371/journal.ppat.1005814.

Jaisinghani, N., Dawa, S., Singh, K., Nandy, A., Menon, D., Bhandari, P.D., et al. (2018). Necrosis Driven Triglyceride Synthesis Primes Macrophages for Inflammation During Mycobacterium tuberculosis Infection. *Front Immunol* 9**,** 1490. doi: 10.3389/fimmu.2018.01490.

Johansen, M.D., de Silva, K., Plain, K.M., Whittington, R.J., and Purdie, A.C. (2019). Mycobacterium avium subspecies paratuberculosis is able to manipulate host lipid metabolism and accumulate cholesterol within macrophages. *Microb Pathog* 130**,** 44-53. doi: 10.1016/j.micpath.2019.02.031.

Johansen, M.D., Hortle, E., Kasparian, J.A., Romero, A., Novoa, B., Figueras, A., et al. (2018). Analysis of mycobacterial infection-induced changes to host lipid metabolism in a zebrafish infection model reveals a conserved role for LDLR in infection susceptibility. *Fish Shellfish Immunol* 83**,** 238-242. doi: 10.1016/j.fsi.2018.09.037.

Kapoor, N., Pawar, S., Sirakova, T.D., Deb, C., Warren, W.L., and Kolattukudy, P.E. (2013). Human granuloma in vitro model, for TB dormancy and resuscitation. *PLoS One* 8(1)**,** e53657. doi: 10.1371/journal.pone.0053657.

Knight, M., Braverman, J., Asfaha, K., Gronert, K., and Stanley, S. (2018). Lipid droplet formation in Mycobacterium tuberculosis infected macrophages requires IFN-gamma/HIF-1alpha signaling and supports host defense. *PLoS Pathog* 14(1)**,** e1006874. doi: 10.1371/journal.ppat.1006874.

Lee, W., VanderVen, B.C., Fahey, R.J., and Russell, D.G. (2013). Intracellular Mycobacterium tuberculosis exploits host-derived fatty acids to limit metabolic stress. *J Biol Chem* 288(10)**,** 6788-6800. doi: 10.1074/jbc.M112.445056.

Mattos, K.A., D'Avila, H., Rodrigues, L.S., Oliveira, V.G., Sarno, E.N., Atella, G.C., et al. (2010). Lipid droplet formation in leprosy: Toll-like receptor-regulated organelles involved in eicosanoid formation and Mycobacterium leprae pathogenesis. *J Leukoc Biol* 87(3)**,** 371-384. doi: 10.1189/jlb.0609433.

Mattos, K.A., Lara, F.A., Oliveira, V.G., Rodrigues, L.S., D'Avila, H., Melo, R.C., et al. (2011). Modulation of lipid droplets by Mycobacterium leprae in Schwann cells: a putative mechanism for host lipid acquisition and bacterial survival in phagosomes. *Cell Microbiol* 13(2)**,** 259-273. doi: 10.1111/j.1462-5822.2010.01533.x.

Peyron, P., Vaubourgeix, J., Poquet, Y., Levillain, F., Botanch, C., Bardou, F., et al. (2008). Foamy macrophages from tuberculous patients' granulomas constitute a nutrient-rich reservoir for M. tuberculosis persistence. *PLoS Pathog* 4(11)**,** e1000204. doi: 10.1371/journal.ppat.1000204.

Podinovskaia, M., Lee, W., Caldwell, S., and Russell, D.G. (2013). Infection of macrophages with Mycobacterium tuberculosis induces global modifications to phagosomal function. *Cell Microbiol* 15(6)**,** 843-859. doi: 10.1111/cmi.12092.

Singh, V., Jamwal, S., Jain, R., Verma, P., Gokhale, R., and Rao, K.V. (2012). Mycobacterium tuberculosis-driven targeted recalibration of macrophage lipid homeostasis promotes the foamy phenotype. *Cell Host Microbe* 12(5)**,** 669-681. doi: 10.1016/j.chom.2012.09.012.

Vrieling, F., Wilson, L., Rensen, P.C.N., Walzl, G., Ottenhoff, T.H.M., and Joosten, S.A. (2019). Oxidized low-density lipoprotein (oxLDL) supports Mycobacterium tuberculosis survival in macrophages by inducing lysosomal dysfunction. *PLoS Pathog* 15(4)**,** e1007724. doi: 10.1371/journal.ppat.1007724.
